# Semiquantitative Screening of THC Analogues by Silica Gel TLC with an Ag(I) Retention Zone and Chromogenic Smartphone Detection

**DOI:** 10.1021/acs.analchem.2c01627

**Published:** 2022-09-30

**Authors:** Si Huang, Ruiying Qiu, Zhengfa Fang, Ke Min, Teris A. van Beek, Ming Ma, Bo Chen, Han Zuilhof, Gert IJ. Salentijn

**Affiliations:** † Key Laboratory of Phytochemical R&D of Hunan Province and Key Laboratory of Chemical Biology & Traditional Chinese Medicine Research of Ministry of Education, Hunan Normal University, Changsha410081, China; ‡ Laboratory of Organic Chemistry, Wageningen University, Wageningen6708 WE, The Netherlands; § Wageningen Food Safety Research (WFSR), Wageningen University & Research, Wageningen6700 AE, The Netherlands; ∥ Department of Chemical and Materials Engineering, Faculty of Engineering, King Abdulaziz University, Jeddah21589, Saudi Arabia

## Abstract

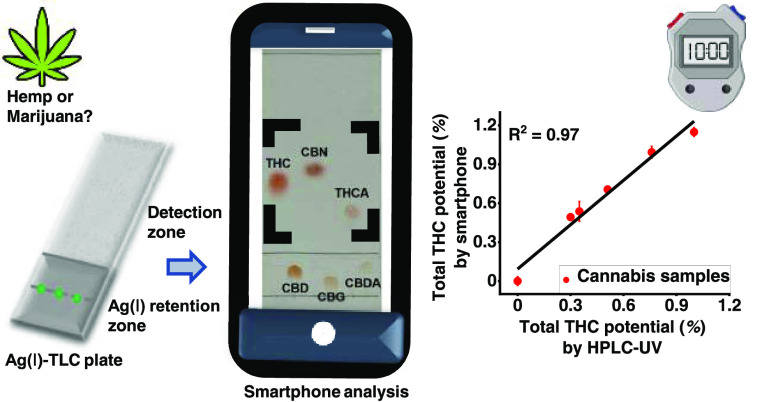

With the ever-evolving cannabis industry, low-cost and high-throughput analytical methods for cannabinoids are urgently needed. Normally, (potentially) psychoactive cannabinoids, typically represented by Δ9-tetrahydrocannabinol (Δ9-THC), and nonpsychoactive cannabinoids with therapeutic benefits, typically represented by cannabidiol (CBD), are the target analytes. Structurally, the former (tetrahydrocannabinolic acid (THCA), cannabinol (CBN), and THC) have one olefinic double bond and the latter (cannabidiolic acid (CBDA), cannabigerol (CBG), and CBD) have two, which results in different affinities toward Ag(I) ions. Thus, a silica gel thin-layer chromatography (TLC) plate with the lower third impregnated with Ag(I) ions enabled within minutes a digital chromatographic separation of strongly retained CBD analogues and poorly retained THC analogues. The resolution (*R*_s_) between the closest two spots from the two groups was 4.7, which is almost 8 times higher than the resolution on unmodified TLC. After applying Fast Blue BB as a chromogenic reagent, smartphone-based color analysis enabled semiquantification of the total percentage of THC analogues (with a limit of detection (LOD) of 11 ng for THC, 54 ng for CBN, and 50 ng for THCA when the loaded volume is 1.0 μL). The method was validated by analyzing mixed cannabis extracts and cannabis extracts. The results correlated with those of high-performance liquid chromatography with ultraviolet detection (HPLC-UV) (*R*^2^ = 0.97), but the TLC approach had the advantages of multi-minute analysis time, high throughput, low solvent consumption, portability, and ease of interpretation. In a desiccator, Ag(I)-TLC plates can be stored for at least 3 months. Therefore, this method would allow rapid distinction between high and low THC varieties of cannabis, with the potential for on-site applicability.

Cannabis (*Cannabis sativa* L.) has been used by man since the stone age as a source of food, fiber, medicine, and psychoactives. It contains a specific class of phytochemicals called cannabinoids.^1^ The most abundant cannabinoids are THCA and CBDA, derived from the same precursor cannabigerolic acid (CBGA) with THCA synthase and CBDA synthase, respectively. These acidic cannabinoids are rather unstable and may lose CO_2_ by exposure to light, air, or heat to produce cannabidiol (CBD), Δ9-tetrahydrocannabinol (THC), and cannabigerol (CBG) with different bioactivities.^2^ Further degradation can occur, for example, cannabinol (CBN) as an oxidative degradation product of THC is found in aged cannabis.^3^ Chemically, these cannabinoids can be roughly divided into two groups: (1) THC analogues that have a pyran ring and (2) CBD analogues that have a disubstituted double bond and a second phenolic group instead of the pyran ring. THCA is not psychoactive, but it can be converted to THC, the main psychoactive cannabinoid, during smoking or baking.^4^ CBN is also psychoactive but has only 10% of the potency of THC.^5^ Congeners of the other group like CBG, CBDA, and CBD are not psychoactive but possess various therapeutic properties (Figure 1).^6^

**Figure 1:**
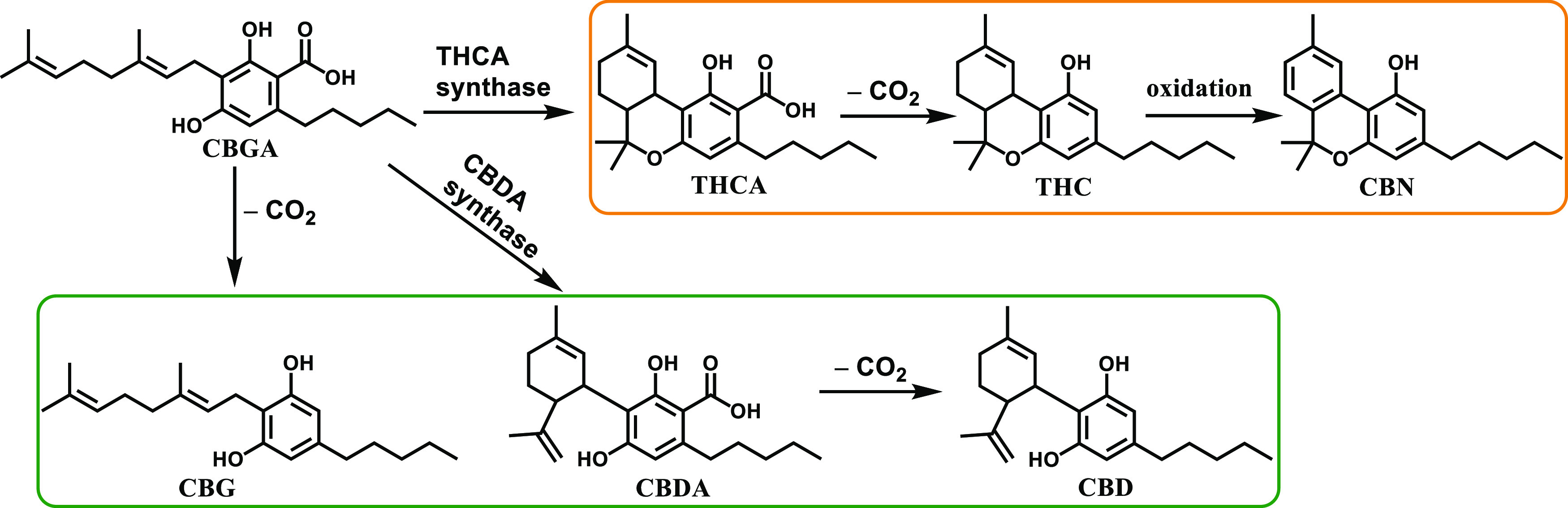
Structures and formation process of important cannabinoids: cannabigerolic acid (CBGA), tetrahydrocannabinolic acid (THCA), Δ9-tetrahydrocannabinol (Δ9-THC), cannabinol (CBN), cannabigerol (CBG), cannabidiolic acid (CBDA), and cannabidiol (CBD).

Although there are many classification systems based on the content of psychotropic components, the drug-type (marijuana) and the fiber-type (hemp) are the most important cannabis types for forensic and legislative considerations. Marijuana is characterized by a high content of the psychoactive compound THC (>0.3% of dry weight).^7^ Given that CBN is a degradation product of THC, it can be included as a relevant parameter when evaluating the initial THC concentration.^8^ For assessing the total THC potential of cannabis, both its precursor THCA and degradation product CBN should thus be taken into consideration.^9,10^ In contrast to marijuana, hemp is characterized by nonpsychoactive cannabinoids including CBDA and its decarboxylated form, namely, CBD, as well as some minor cannabinoids, e.g., CBGA and CBG.^11^ Since THCA, CBDA, and CBG are all formed from the common precursor CBGA,^12^ many cannabis plants contain THCA, CBDA, and CBG at the same time. Moreover, in many screening and instrumental analyses, it is difficult to differentiate between different cannabinoids from those different groups. As a result, the separation of CBD analogues from THC analogues is necessary to prevent interference.

The growing cannabis industry and increasing pressure on forensic testing have pushed the development of portable, high-throughput, and easy-to-use tests that can be performed directly in the field for qualitative and quantitative analysis of cannabis.^13^ Kurouski et al.^14,15^ proposed a noninvasive and nondestructive method to distinguish between freshly frozen plants rich in THCA and rich in CBD using a handheld Raman spectrometer. Due to the variation in intensities of characteristic vibrational bands from cellulose, xylan, carotenoids, lignin, and THCA in different plant materials, this method could be used to classify cannabis varieties. However, this method only enables qualitative analysis by comparison with previously collected Raman spectroscopic signatures from various cannabis varieties. Valid quantification of individual cannabinoids still needs further study. Arce et al.^16^ applied thermal desorption (TD)-ion mobility spectrometry (IMS) to analyze dried leaves and flowers of plant samples for potentially on-site discrimination of cannabis varieties. However, most signals in the TD-IMS spectra under both positive and negative ionization modes could not be clearly identified.

Compared with the above-mentioned instrument-dependent in-field screening methods, colorimetric tests are attractive alternatives, as they are cheap and require minimal operational training. Colorimetric test kits based on fast blue BB salt or Duquenois–Levine reagent are commonly used by forensic laboratories for the analysis of marijuana samples. However, without any pre-separation, false-positive results can be obtained from nonpsychoactive cannabinoids, e.g., CBD analogues, as well as noncannabinoids.^17^ Therefore, separation of individual cannabinoids before color development is a necessity.

To achieve such separation, the combination of thin-layer chromatography (TLC) and a chromogenic reagent for visualization of spots has been demonstrated, leading to simple, fast, and qualitative or semiquantitative analysis of cannabis. Different stationary phases,^18,19^ solvent systems,^13^ and methods for semiquantitative image analysis have been tested.^20^ Liu et al.^13^ evaluated different mobile phases for analysis of possibly illegal cannabis products on silica gel and a reversed-phase (C-18) plate. Both systems provided good separation for Δ9-THC, CBD, and CBN in hemp and marijuana samples but could not adequately separate the acidic cannabinoids like THCA, CBDA, and CBGA. In other studies, the stationary phase has been modified to obtain better separations.^18,19^ Tsujikawa et al.^19^ recently analyzed five THC isomers (Δ9-THC, Δ8-THC, a pair of diastereomers of Δ10-THC, and Δ6a,10a-THC), CBD, CBN, and THCA in samples of CBD heated in acidic ethanol on silica gel TLC plates modified with 10% AgNO_3_ and toluene as solvent. After chromogenic detection, this system resolved Δ9-THC, CBD, CBN, and Δ8-THC relying on the specific affinity of Ag(I) toward compounds with different numbers or different positions of olefinic double bonds, which has been described in previous work.^21−23^ This mechanism has also been used in our recent work for the differentiation of isomers THC and CBD by Ag(I)-impregnated paper spray mass spectrometry.^24^ However, a disadvantage of the method developed by Tsujikawa et al. was that the THCA spot was tailing and overlapped with the CBD spot. Considering this overlap and the fact that Δ8-THC is not a target compound for analysis of herbal cannabis, the authors explicitly stated that this system is not suitable for cannabis plant samples. Furthermore, because the entire TLC plate was modified by AgNO_3_, significant background color from the reaction of Ag(I) ions with the basic ammonia solution interfered with the detection of cannabinoids.

In addition to the use of a TLC for the separation of different cannabinoids, the listed applications are based on the use of chromogenic reagents for qualitative analysis. For TLC-based quantitative analysis, research has relied on using a photoelectric densitometer to scan spots,^20,25,26^ which is not suitable for instrument-free on-site analysis. The portability, low cost, versatility, and wide availability of smartphones create opportunities for the instrument-free analysis of TLC plates. Scientific efforts have focused on smartphone-based qualitative and quantitative analyses and led to applications in the detection of (heavy) metals, herbicides, pesticides, antibiotics, biochemical indicators, allergens, bacteria, viruses, and so on.^27−29^

The aim of this research was to improve both the TLC separation of THC analogues using a partially modified silica gel TLC plate with Ag(I) ions and the detection step by semiquantitatively scanning the colored THC spots with a smartphone. This combination of techniques would allow for a fast, instrument-free, in-field screening of cannabis varieties in a greenhouse setting or for on-site forensic purposes with a low solvent consumption.

## Materials and Methods

### Overview of the Experiments

**Figure 2:**
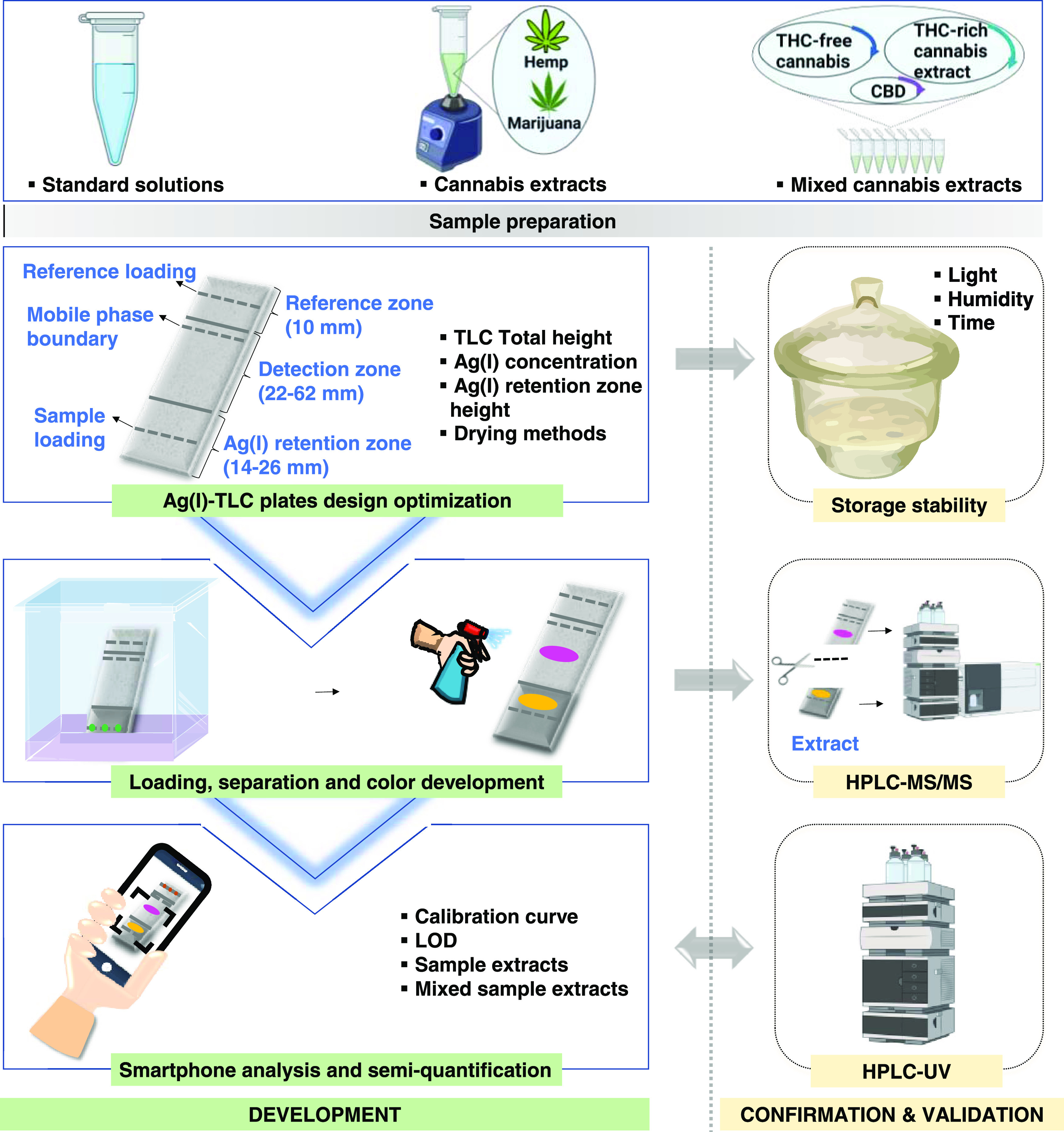
Overview of the experimental workflow (created partly with biorender.com).

The aim of this work is to semiquantify psychoactive cannabinoids with a fast and portable tool. To achieve this, the experimental design, given in Figure 2, was followed. This started with sample preparation, including standard solutions, cannabis extracts, and mixed cannabis extracts. Next, Ag(I)-TLC plates were designed and optimized for the digital chromatographic separation of THC analogues from CBD analogues, and their storage stability was investigated with respect to light, humidity, and time. Following that, samples were loaded on the optimized Ag(I)-TLC plates and went through the separation and color development process. The quality of the separation was then validated by HPLC-MS/MS, by recovering cannabinoids from different regions of the TLC plates, and analyzing those. Subsequently, calibration curves were constructed, based on smartphone analysis of the colored spots on the TLC plate, and the LODs of THCA, THC, and CBN were evaluated. Finally, the total THC potential percentages of cannabis extracts and mixed cannabis extracts were semiquantified by the developed smartphone analysis and benchmarked against HPLC-UV.

### Chemicals and Reagents

Acetonitrile (ACN, HPLC-grade) and methanol (MeOH; HPLC-grade) were purchased from Sigma-Aldrich (St. Louis). Hexane (HPLC-grade), *tert*-butyl methyl ether (MTBE; HPLC-grade), and acetic acid (AcOH; HPLC-grade) were purchased from Sinopharm Chemical Reagent Co., Ltd. (Shanghai, China). Deionized water was prepared by a Milli-Q water purification system (Millipore). AgNO_3_ (AR, 99.8%) and NaOH (AR, 99.8%) were purchased from Aladin Biochemical Technology Co. Ltd. (Shanghai, China). 4-Benzoylamino-2,5-diethoxybenzenediazonium chloride hemi (zinc chloride) salt (Fast Blue BB; FBBB; AR grade) was obtained from Sigma-Aldrich (St. Louis). Olivetol (5-pentylbenzene-1,3-diol, AR, 99.7%) was bought from Shanghai Haohong Biomedical Technology Co., Ltd. (Shanghai, China). Aluminum-backed silica gel 60 F_254_ 20 × 20 cm^2^ TLC plates were purchased from Merck (Darmstadt, Germany). THCA (99.2%), THC (99.0%), CBN (99.2%), CBDA (99.3%), CBD (99.8%), and CBG (99.1%) standards were obtained from Henan Wanjia Reference Material R&D Center Co., Ltd. (Zhengzhou, China). Pure CBD powder (99.5%) and 10% (w/w%) CBD oil (hemp seed oil as matrix) were bought from Yuxi Hongbao Biological Technology Co., Ltd. (Yunnan, China). Dry plant materials were purchased online in China, and are referred to as cannabis_1, cannabis_2, and cannabis_3. Marijuana extract was prepared from marijuana inflorescence and concentrated by the evaporation of most solvent.

### HPLC-(PDA) MS/MS Setup

A high-performance liquid chromatograph (HPLC Prominence; Shimadzu, Kyoto, Japan), equipped with an Ultimate LP-C18 column (4.6 × 150 mm^2^, 5 μm; Welch Materials, Inc., West Haven) and an SPD-M20A photodiode array detector was coupled to a triple-quadrupole mass spectrometer (MS8050; Shimadzu, Kyoto, Japan). The mobile phase consisted of 0.1% (v/v%) formic acid in both water (mobile phase A) and acetonitrile (mobile phase B), and the flow rate was 1.0 mL·min^–1^: 0–12 min 80% B; 12–13 min linear ramping to 100%; 13–18 min 100% B; 18–19 min linear decrease to 80% B; 19–28 min reequilibration at 80% B. Mass spectrometry was performed in positive multiple reaction monitoring (MRM) mode (see Table S3 for settings) with an atomizer flow rate of 3 L·min^–1^, a heating gas flow rate of 10 L·min^–1^, a drying gas flow rate of 10 L·min^–1^, DL temperature 250 °C, ion source interface voltage of 4 kV, and heating block temperature of 400 °C.

### Sample Preparation

#### Standard Solutions

THCA, THC, CBN, CBDA, CBD, and CBG methanol stock solutions (1.00 μg·μL^–1^) were diluted with MeOH to obtain a series of standard solutions for subsequent analysis. A “standard_mixture” was prepared containing 83.3 ng·μL^–1^ THCA, THC, CBN, CBG, CBDA, and CBD in MeOH.

#### Cannabis Extracts

MeOH (300 μL) was added to 100.0 mg of dried, homogenized, and ground herbal cannabis_1, cannabis_2, and cannabis_3 one by one with a pipette (Eppendorf 3120000267, 100–1000 μL). The extraction was performed by vortexing for 3 min with Vortex-Genie 2 (Scientific Industries, Inc.). The extracted samples were filtered using a 0.2 μm PTFE membrane syringe filter (⌀13 mm). Marijuana extract concentrate was diluted with MeOH to 10.00 and 1.00 mg·mL^–1^. 10% CBD oil was diluted with MeOH 50-fold. All solutions except for 10.00 mg·mL^–1^ marijuana extract concentrate were analyzed by HPLC-UV at 228 nm for quantification of six cannabinoids according to external standard calibration curves (Figure S1 and Table S1). A “sample_mixture” was prepared by mixing 60.0 μL of cannabis_1 extract, 120.0 μL of cannabis_2 extract, 60.0 μL of cannabis_3 extract, 60.0 μL of marijuana extract, and 60.0 μL of CBD oil extract.

#### Mixed Cannabis Extracts

Different volumes of marijuana extract (high levels of THC analogues) were spiked into cannabis_3 plant material (no THC analogues, blank matrix) to produce samples containing different concentrations of THC analogues (mixed cannabis extracts set I, Table S2). Next, different volumes of marijuana extract (high levels of THC analogues) and 1.00 mg of CBD powder were spiked into cannabis_3 plant material (no THC analogues, blank matrix) to produce samples containing both THC analogues and CBD (mixed cannabis extracts set II, Table S2).

### Ag(I)-TLC Plates Design, Optimization, and Storage Stability

#### Ag(I)-TLC Plate Design

The 20 × 20 cm^2^ aluminum-backed silica gel TLC plates were cut with scissors into rectangular plates. The plates were made with three regions: (1) Ag(I) retention zone, (2) detection zone, and (3) reference zone, as shown in Figure 2 (see Figure S2 for more details).

#### Ag(I)-TLC Plate Optimization

THCA, THC, CBN, CBDA, CBD, and CBG standard solutions were analyzed (see protocol below) and the resolution between the pairs THCA-CBD, THC-THCA, and THC-CBN were investigated to optimize (i) the total height of the TLC plate, (ii) AgNO_3_ concentration for TLC modification, (iii) Ag(I) retention zone height, and (iv) drying method of modified TLC plates step by step (see Protocol S1 for details).

#### Ag(I)-TLC Plates Storage Stability

A batch of Ag(I)-TLC plates was prepared and stored in sealed plastic bags with the aluminum side up, under three different conditions: on a desk, in a black box, and in a brown desiccator with anhydrous calcium chloride as a drying agent at room temperature (Figure S3). At regular intervals, three pieces of Ag(I)-TLC plates under each storage condition were tested with THC and CBD standards to evaluate the separation performance over time.

### Sample Loading, Separation, and Color Development

#### Sample Loading and Separation

To load a sample on the TLC plate, 1.0 μL of an extract solution was applied on both the sample loading line and the reference sample loading line of the TLC plate with a pipette (Eppendorf 3120000216, 0.1–2.5 μL). After drying, the TLC plate was placed into a sealed chamber (width 4 cm, height 10.5 cm, with removable lid) conditioned with mobile phase (MTBE/hexane = 1:4 v/v with 0.1% AcOH) vapor for at least 5 min with a piece of filter paper on the wall of the tank to facilitate the equilibration.^30^ When the mobile phase reached the mobile phase boundary line, the TLC plate was taken out and dried in a fume hood. The loaded samples on the reference sample line were not eluted by the mobile phase but were included in the color development procedure.

#### Color Development

The color developing procedure needs A and B solutions consisting of 0.2 M NaOH solution in water/MeOH (1:9 v/v) and a 5 mg·mL^–1^ FBBB MeOH solution, respectively. A and B solutions were ready for use in glass spray bottles (Figure S4). For color development, solution A was evenly sprayed by applying five pumps on the developed TLC plate, and then solution B was evenly sprayed by applying 10 pumps. After 2 min, the TLC plate was moved to a custom-made light box (Figure S5) and an Apple iPhone 11 camera was used for image acquisition.

#### Confirmation by HPLC-MS/MS

Extracts were made of the scraped-off silica from the different regions of the TLC plates, and these were evaluated by HPLC-MS/MS to (i) confirm the identity of the cannabinoids present in the different regions, (ii) to quantify those cannabinoids to assess the separation efficiency, and (iii) to evaluate whether other cannabinoids are present at low levels that are missed by the colorimetric analysis. The detailed procedures can be found in Protocol S2. In short, the “standard_mixture” and “sample_mixture” were analyzed by both unmodified TLC plate and Ag(I)-TLC plate. The silica gel in the upper part and lower part of each plate was scraped off separately and extracted with MeOH for HPLC-MS/MS analysis of separated cannabinoids.

### Smartphone Analysis and Semiquantification

#### Calibration Curve Construction

To achieve semiquantification of major psychoactive cannabinoids with smartphone analysis, calibration curves of THCA, THC, and CBN were constructed and the LODs were evaluated. THCA, THC, and CBN methanol solutions (0.20, 0.50, 0.80, and 1.00 μg·μL^–1^) (1.0 μL) were loaded on the sample loading line of an Ag(I)-TLC plate, and 1.0 μL of a 1.00 μg·μL^–1^ solution of each analyte was loaded twice to have an absolute amount of 2.00 μg. Each solution was analyzed on three different TLC plates. Olivetol (1.0 μL of 0.10 μg·μL^–1^ in MeOH loaded on a square TLC plate) was chosen as reference to correct for variation in color development and image acquisition because it also reacts with FBBB to form a colored product. ImageJ (NIH) software was used to obtain three image parameters, namely, hue (H), saturation (S), and brightness (B).^27^ Each spot on the image was analyzed, and the obtained value for saturation was normalized against the obtained saturation value of the olivetol reference spot. The normalized saturation values for THCA, THC, and CBN were plotted as a function of their concentration.

#### Evaluation of LOD

The LOD was calculated as follows: LOD = 3× SD of blank/slope of curve, in which the blank is the normalized background saturation of analyzed TLC plates. Additionally, to check whether the calculated LODs match with actual observations by the naked eye, 1.0 μL of 62.5, 31.3, 15.6, and 7.8 ng·μL^–1^ of THCA, THC, and CBN was loaded on Ag(I)-TLC plate and went through solvent-development as well as color development processes.

#### Analysis of (Mixed) Cannabis Extracts

(Mixed) cannabis extracts (1.0 μL) (Table S2) were analyzed by Ag(I)-TLC plates with smartphone detection. The normalized THCA saturation value was used to calculate THCA content in samples by the above-constructed THCA calibration curve. The normalized THC and CBN saturation value was used to calculate the sum content of THC + CBN in samples by the above-constructed THC calibration curve unless noted otherwise. Further image analysis in RGB space was explored to assess whether the color of the THC and CNB spot could be used to assess whether it is high in THC or CBN, as pure spots produce purple and orange spots, respectively. Subsequently, total THC potential percentage was expressed as follows
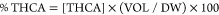
%THC and %CBN were calculated similarly.
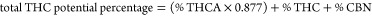
where [THCA] is the measured concentration of THCA (μg/mL), VOL is the external volume (300 μL), DW is the dry sample weight (100.0 mg), and 0.877 is the molecular weight ratio of cannabinoids to cannabinoid acids under study.^31,32^

#### Validation by HPLC-UV

The concentration of THCA, THC, and CBN in cannabis extracts was determined by external standard calibration curves (Figure S1 and Table S1) with HPLC-UV at 228 nm. The total THC potential percentages in (mixed) cannabis extracts were calculated in the same way as for smartphone analysis except for the following difference in mixed cannabis extracts.


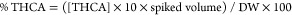


[THCA] (μg·mL^–1^) is the measured concentration in 1.00 mg·mL^–1^ marijuana solution from HPLC-UV at 228 nm, and the spiked volume is 0, 0.12, 0.14, 0.2, 0.3, 0.4, 0.5 or 0.6 mL, DW is the dry sample weight (100.0 mg), and %THC and %CBN were calculated similarly.

## Results and Discussion

### Comparison of Ag(I)-TLC and Unmodified TLC for Separation of Standards

As colored products are formed by coupling FBBB to the para position relative to the phenolic group in slightly alkaline solution, the colorimetric reaction is highly selective for phenols, but does not distinguish individual members within the cannabinoid group.^33^ There are many varieties of cannabis containing both THC analogues and CBD analogues, which are difficult to separate by traditional TLC methods.^34^ The difference in affinity of Ag(I) ions with a single alkene C=C bond (weak) versus a 1,5-diene (strong)^21^ was exploited here to allow the separation of these two groups.

**Figure 3:**
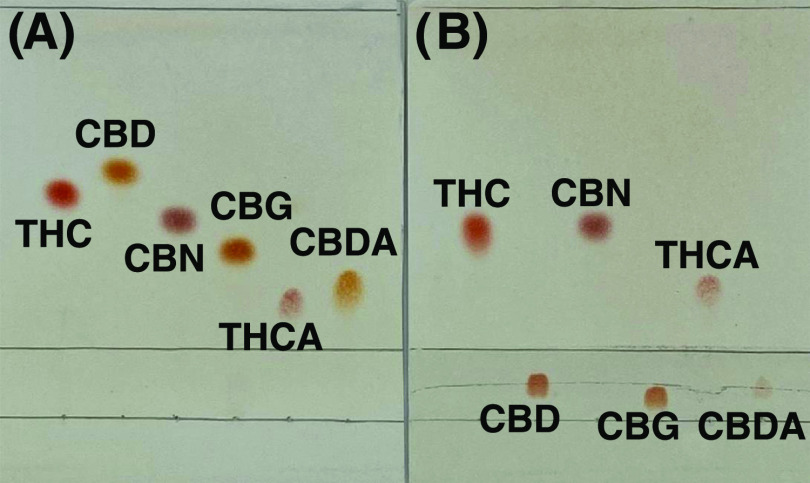
THC, CBD, CBN, CBG, THCA, and CBDA standards (from left to right) loaded and separated on (A) an unmodified silica gel TLC plate and (B) an Ag(I)-TLC plate.

As shown in Figure 3A, when applying unmodified TLC, THC and CBD (isomers) have similar *R*_f_ values (0.55 and 0.60, respectively) as well as the pair THCA and CBDA (isomers) (0.29 and 0.34, respectively). The resolution of the closest two spots (one from THC analogues and the other one from CBD analogues) was only 0.6, making it difficult to distinguish the THC analogues and CBD analogues, which is necessary for assessing the total THC potential without interference from CBD analogues. However, after extensive optimization (Figures S6 and S7), a partially coated Ag(I)-TLC plate was successfully developed and could be used to separate CBD analogues from THC analogues (Figure 3B). The resolution between the highest CBD analogue and the lowest THC analogue was 4.7, which is almost 8 times the resolution on an unmodified TLC plate.

### Storage Stability of Ag(I)-TLC Plates

**Figure 4:**
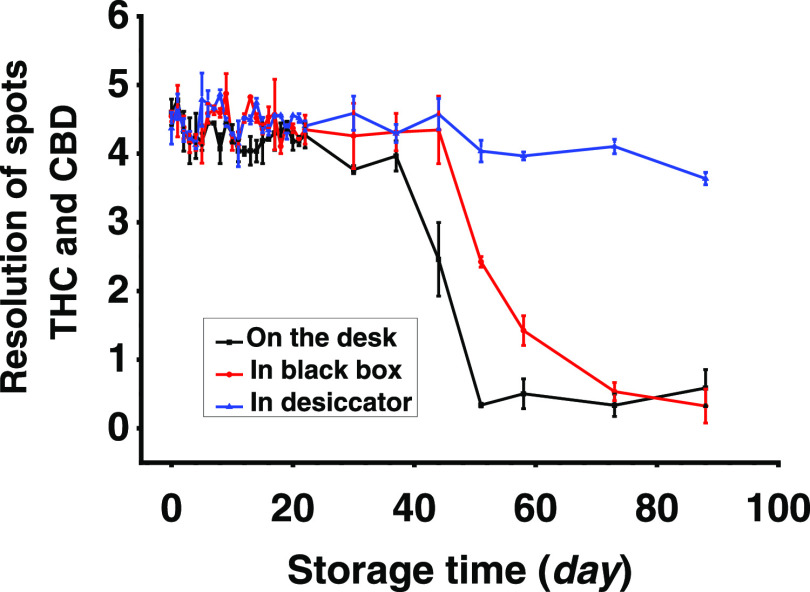
Changes in resolution between THC and CBD during storage in plastic sealed bags and put on a desk, in a black box, or in a brown desiccator. Error bars represent standard deviation (*n* = 3).

After obtaining the optimized Ag(I)-TLC plates, storage stability was investigated for robust applicability, considering the sensitivity of Ag(I) toward light and humidity (Figures 4 and S8).^35^ There is little difference in separation between THC and CBD for the first 37 days between plates stored under different conditions. This is probably because all Ag(I)-TLC plates were put in plastic, sealed bags with the aluminum side up so that the influence of light and humidity could be somewhat prevented during the initial storage stage. With extended storage time, however, the separation performance of Ag(I)-TLC plates stored on the desk dropped sharply. During this process, the Ag(I) retention zone became visibly and increasingly darker due to the photodegradation of Ag(I) (Figure S8), which resulted in the compromised retention effect toward CBD. Moreover, the absolute *R*_f_ of THC and CBD increased (Figure S9), possibly due to the competitive binding of water molecules on silica. When these plates are stored in a black box, the effect from light can be excluded, and the separation quality was constant for 44 days but did eventually deteriorate upon storage between 44 and 88 days. Storage in the brown desiccator with a drying agent yielded the best result, as separation performance remained good during the entire stability study, which lasted 88 days. Therefore, when suitably stored, mass-produced Ag(I)-TLC plates could be used for large-scale application.

### Assessment of Ag(I)-TLC Separation by HPLC-MS/MS

**Figure 5:**
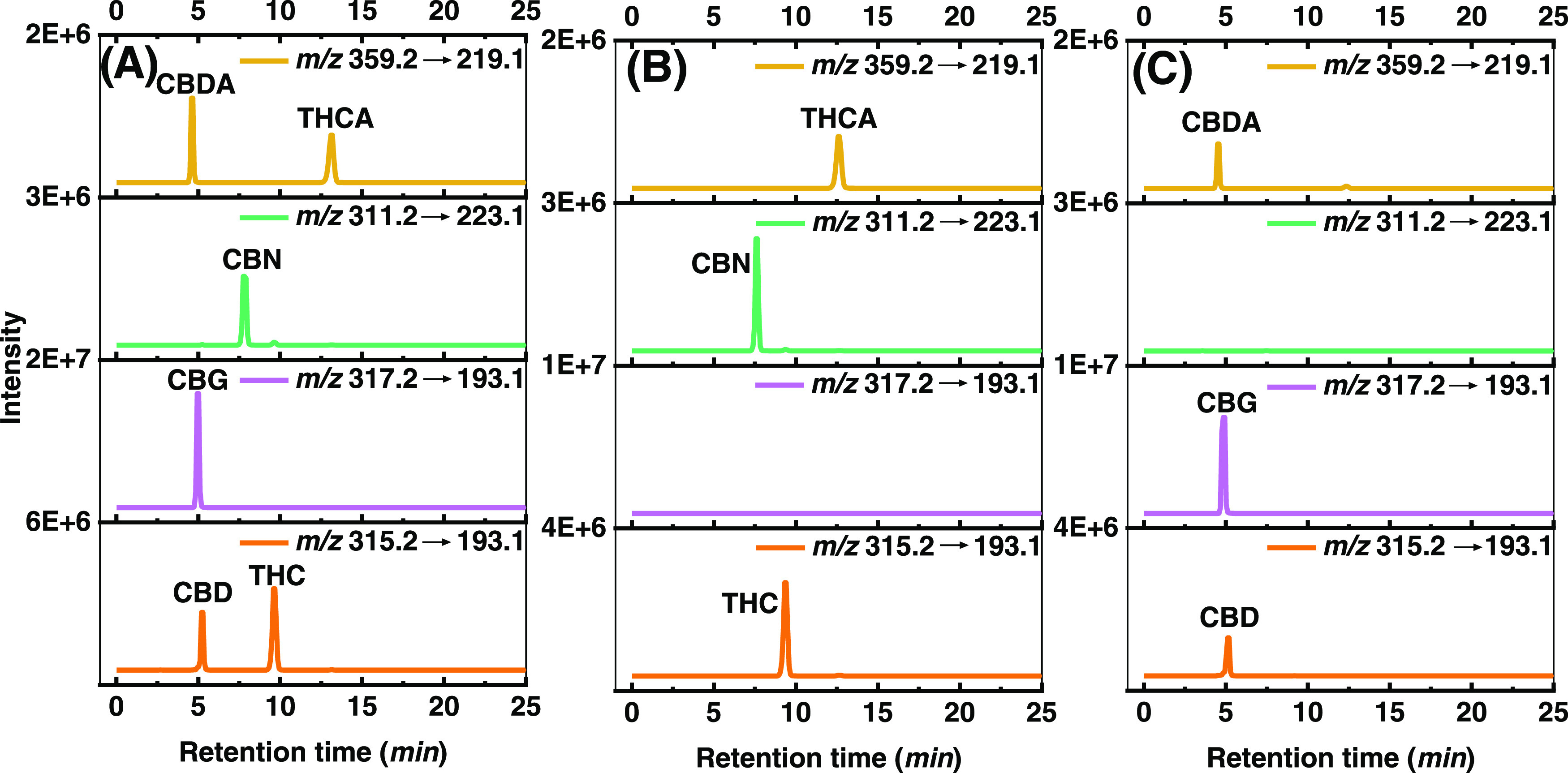
HPLC-MRM chromatograms of detected cannabinoids in (A) “standard_mixture,” (B) extract of Ag(I)-TLC detection zone silica after separating “standard_mixture,” and (C) extract of Ag(I) retention zone silica after separating “standard_mixture”.

After obtaining a digital chromatographic separation (Figure S10), HPLC-MS/MS was used to identify and quantify the cannabinoids in the upper and lower parts of the TLC plate, in the “standard_mixture” and “sample_mixture.” THCA, CBDA, THC, CBD, CBN, and CBG were identified by comparing with standards to match retention time and MRM signals (see Figures 5A and S11). Other cannabinoids found in sample extracts were identified by matching MRM signals and relative retention times with the literature (Figures S12 and S13).^36−38^ THC, CBN, and THCA were identified in the detection zone, while CBD, CBG, and CBDA were detected in the retention zone of the Ag(I)-TLC plate (Figure 5B,C), which is consistent with the observed colorimetric results. In contrast, when applying unmodified silica TLC, all of these six standards were found in the detection zone (Figure S11). During the HPLC-MS/MS analysis of the “sample_mixture,” 12 cannabinoids could be identified. On unmodified silica TLC, signals of all 12 cannabinoids were found in the upper part, and most cannabinoids, except CBDV, CBG, and CBDA, were also found in the bottom part (Figure S12). For Ag(I)-TLC plates, all THC analogues (THCV, THC, CBN, THCVA, THCA, CBLA) were found in the upper part (detection zone) and there were no signals of any CBD analogues (CBDA, CBD, CBG, CBDVA, CBDV, CBGA); in the bottom part (Ag(I) retention zone), the main signals were from the CBD analogues (CBDV, CBD, CBG, CBDVA, CBDA, and CBGA) (Figure S12). Two minor signals of THCVA and THCA were also found in the Ag(I) retention zone. However, the peak area of THCVA only accounts for 4.0 ± 1.0% of the corresponding signal peak area found in the upper part (detection zone), while for THCA the value is 4.7 ± 1.0%. The retention of these two acidic compounds in the bottom part is also found on normal TLC plates, probably due to the interaction between the carboxyl moieties and silica-based silanol.

Based on the colorimetric detection and mass spectrometric identification of cannabinoids separated with Ag(I)-TLC plate, it can be seen the Ag(I)-TLC plate has the ability to divide cannabinoids into two groups. Structurally, in the bottom part (Ag(I) retention zone), all cannabinoids share a 1,5-diene moiety, while the cannabinoids in the detection zone have no or only one double olefinic bond (Figure S13). From the perspective of psychoactive effects, two compounds are most relevant: THCV, which is the decarboxylated product resulting from (nonpsychoactive) THCVA, and possesses ∼25% of the psychoactive potency of THC,^39^ while CBLA is a rare and poorly investigated cannabinoid, whose decarboxylated product CBL was initially named “THC III”.^40^ For the CBD analogues, CBD (decarboxylated from CBDA), CBG, and CBDV (decarboxylated from CBDVA) are not psychoactive and have various therapeutic properties. Thus, it appears that the cannabinoids found in the detection zone tend to be (potentially) psychoactive, while those retained in the Ag(I) retention zone are nonpsychoactive.

### Ag(I)-TLC Coupling with Smartphone for Semiquantitative Analysis of THC Analogues

After fully assessing the separation performance of Ag(I)-TLC plates, smartphone analysis was used for semiquantification of THC analogues.

#### Calibration Curves and LOD

Calibration curves between the absolute amount of THCA, THC, or CBN loaded on the plate and the saturation values were first constructed (Figure S14), which showed good linearity (*R*^2^ = 0.96–0.98) in the range of 0.2–2 μg and LODs of 11 ng for THC, 54 ng for CBN, 50 ng for THCA. The lowest visible amount on a developed TLC plate was 15.6 ng for THC, 31.3 ng for CBN, and 31.3 ng for THCA (see Figure S15), which are similar to the calculated values.

#### Distinction between THC and CBN by Image Analysis

By applying the smartphone calibration curves, separate semiquantitative analysis of THCA in samples can be achieved. However, since the spots of THC and CBN on Ag(I)-TLC plates overlap (Figure 3B), separate analysis of THC and CBN is challenging. In a previous study, the sum of THC + CBN has been used to assess the initial THC level and thus indirectly cannabis potency.^32^ Similarly, in this work, the combined THC and CBN spot was used to estimate the total amount of THC + CBN from the THC calibration curve. Due to the slightly different smartphone signal contributions from individual THC and CBN (as shown in their separate calibration curves in Figure S14), some error can be expected when analyzing samples containing various relative compositions of THC and CBN. To investigate this, a series of THC + CBN standard mixtures with same absolute amount but different CBN/(THC + CBN) ratios were analyzed by the Ag(I)-TLC plates (Figure S16A), and the smartphone saturation signal from each spot was compared with that from the pure THC spot. When the CBN/(THC + CBN) ratio is between 0 and 0.6, the saturation signals showed little differences with that from pure THC; however, when the CBN/(THC + CBN) ratio is larger than 0.6, saturation signals accounted for around 70% of the signal from pure THC (Figure S16B). Under this circumstance, applying a THC-only calibration curve to calculate the sum content of THC + CBN would end up with an underestimation of the THC + CBN content. Considering the fact that fresh cannabis or relevant products normally contain little CBN (CBN/(THC + CBN) < 0.6),^32,41^ this would not lead to issues with our semiquantitative method in most cases. However, samples could contain higher CBN content (CBN/(THC + CBN) > 0.6) due to long storage, possibly at ambient temperature or without protection from light.^32^ It was thus investigated whether smartphone image analysis could be used to at least identify when this would be the case. This check is based on the fact that pure THC produces an orange color, whereas pure CBN produces a purple color, after reacting with FBBB. The distinction between such high and low CBN samples could be achieved by RGB color analysis. In short, the relative difference between the R and B components was measured in ImageJ and normalized to a value ranging between 100% THC (1) and 100% CBN (0) (see Figures S17 and S18 for more details). This method allows identification of samples containing higher ratios of CBN/(THC + CBN) (>0.6), for which the application of the CBN calibration curve would then be appropriate.

#### Analysis of (Mixed) Cannabis Extracts

**Figure 6:**
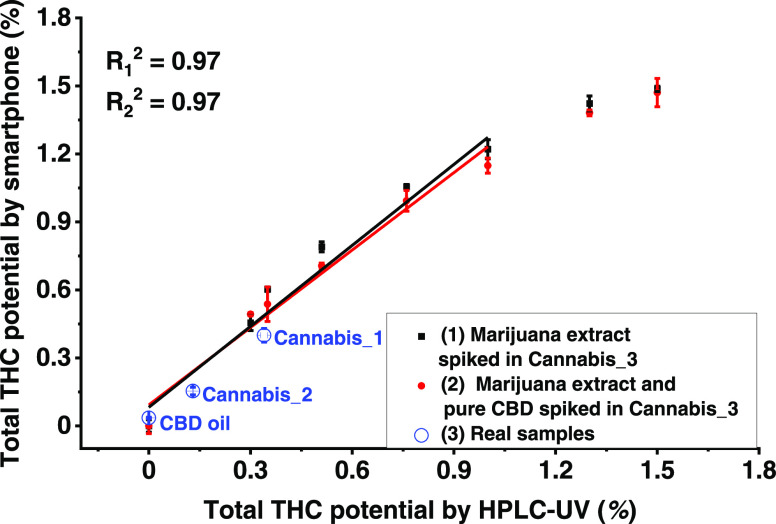
Relationship between total THC potential percentage and normalized saturation of THC analogues.

Mixed cannabis extracts set 1 and mixed cannabis extracts set II (see Table S2) were analyzed by Ag(I)-TLC coupled with smartphone detection (Figure S19A,B). The THC calibration curve was applied to calculate the sum content of THC + CBN since the RGB color analysis showed that the marijuana extract has a CBN/(THC + CBN) ratio smaller than 0.6 (Figures S17 and S18). The total THC potential percentage was calculated and plotted against the spiked total THC potential percentage. As shown in Figure 6, the calculated total THC potential percentages have good correlation with the spiked total THC potential percentages for these two sets of samples (*R*^2^ = 0.97), up to ∼1%. Apart from that, the curves representing two sets of samples almost overlap, which means that there is no obvious interference from CBD for the analysis of THC analogues, due to the digital chromatographic separation by the developed Ag(I)-TLC plate. The results of CBD spiked samples also show the potential application of screening illegal CBD products according to diverse legislative status toward THC (legal limit varies from 0.05 to 1%) and CBN (controlled substance in the U.K.) in CBD-related products among the world.^42,43^ Considering that the Ag(I) retention zone has the ability to retain CBD, CBG, and CBDA up to 10 μg (Figure S20) and the linear range for THC and CBN is 0.2–2 μg, the detectable ratio range of THC/CBD would be limited to 0.02–0.2. However, according to our previous analysis results of commercially available oils labeled as “CBD oil,” four out of 10 CBD oils contained a THC/CBD ratio higher than 0.02,^21^ which means they could also have been detected by this Ag(I)-TLC coupled with a smartphone.

To explore the method for real samples, two cannabis samples and one commercial CBD oil were analyzed as well (Figure S19C). When performing RGB color analysis, cannabis_1 and cannabis_2 gave a result that indicated a CBN/(THC + CBN) ratio higher than 0.6 (Figures S17 and S18), which matches with the HPLC-UV results with a CBN/(THC + CBN) ratio 0.7 for cannabis_1 and 0.9 for cannabis_2 (Table S1). Therefore, the CBN calibration curve instead of THC calibration curve was applied for calculating the sum of THC + CBN. The obtained total THC potential percentages from cannabis_1, cannabis_2, and CBD oil were compared with the HPLC-UV analysis (Figure 6). These results were consistent with HPLC-UV results, although the precision was worse (for HPLC-UV, RSD varies from 1.2 to 3.1%; for smartphone, RSD varies from 5.9 to 10.8%). The calculation of total THC potential in this study may result in an overestimation toward the psychoactive effects of the tested samples considering the lower potency of CBN (around 10%) compared with THC. However, as a screening method, it can minimize false-negative results by reflecting the maximum psychoactive potential.

## Conclusions

The Ag(I)-TLC smartphone method that we describe in this paper allows for a reliable semiquantitative analysis of total THCA, THC, and CBN, which could be potentially applied in the field as a fast screening method for the classification of cannabis varieties. The method is based on a different complexation of THC analogues and CBD analogues with Ag(I) ions, leading in turn to good separation of these cannabinoid classes. As THC analogues migrate out of the Ag(I) area, interference from Ag(I) with the color analysis is avoided. Afterward, smartphone image analysis affords a semiquantitative tool for determining total THC analogues percentage. Furthermore, based on different RGB color information of THC and CBN spots, a distinction between high ratio (>0.6) of CBN/(THC + CBN) and low ratio (≤0.6) of CBN/(THC + CBN) could be achieved. Multiple cannabis herbal samples can be analyzed simultaneously by the developed method within 10 min, requiring only a few milliliters of solvent. The good correspondence between HPLC-UV and the Ag(I)-TLC methods for different samples confirms the applicability of the current method. Samples identified as positive by our cheap and fast screening method can then be re-tested in the lab by a validated quantitative method, such as HPLC-UV. The developed method reinvigorates TLC analysis of THC analogues and requires no special instruments. Thus, it could be useful on-site and affordable to local authorities and small laboratories due to its simplicity, low operating cost, and use of inexpensive and general instruments, and seems an ideal approach to conduct large-scale surveillance programs for the rapid detection and content determination of cannabinoids. Apart from that, this method is also promising to be applied for cannabis freshness identification and THC screening in CBD products.

## Supporting Information Available

Contents of THCA, CBN, THC, CBD, CBG, and CBDA in samples by HPLC-UV and preparation of mixed cannabis extracts; design and optimization of Ag(I)-TLC plates and experimental parameters; images of storage conditions and absolute *R*_f_ of THC and CBD obtained by Ag(I)-TLC plates stored in different conditions and duration; images of color developing and photographing; procedure for extracting cannabinoids from TLC plates; HPLC-MS/MS analysis of separated cannabinoids; chemical structures of detected cannabinoids separated by as Ag(I)-TLC; smartphone calibration curves of THC, CBN, THCA, and CBN/(THC + CBN) ratio; and total THC potential percentage analysis by ImageJ software (PDF)

## References

[ref1] Fischedick J. T. Identification of terpenoid chemotypes among high (−)-trans-Δ9- tetrahydrocannabinol-producing *Cannabis sativa* L. cultivars. Cannabis Cannabinoid Res. 2017, 2, 34–47. 10.1089/can.2016.0040. 28861503 PMC5436332

[ref2] Nachnani R.; Raup-Konsavage W. M.; Vrana K. E. The Pharmacological Case for Cannabigerol. J. Pharmacol. Exp. Ther. 2021, 376, 204–212. 10.1124/jpet.120.000340. 33168643

[ref3] Trofin I.; Vlad C.; Noja V.; Dabija G. Identification and characterization of special types of herbal cannabis. UPB Sci. Bull., Ser. B 2012, 74, 119–130.

[ref4] Radwan M. M.; Chandra S.; Gul S.; ElSohly M. A. Cannabinoids, phenolics, terpenes and alkaloids of cannabis. Molecules 2021, 26, 2774 10.3390/molecules26092774. 34066753 PMC8125862

[ref5] Sharma P.; Murthy P.; Bharath M. M. S. Chemistry, metabolism, and toxicology of cannabis: clinical implications. Iran. J. Psychiatry 2012, 7, 149–156. 23408483 PMC3570572

[ref6] Martínez V.; Iriondo De-Hond A.; Borrelli F.; Capasso R.; del Castillo M. D.; Abalo R. Cannabidiol and other non-psychoactive cannabinoids for prevention and treatment of gastrointestinal disorders: useful nutraceuticals?. Int. J. Mol. Sci. 2020, 21, 3067 10.3390/ijms21093067. PMC7246936 32357565

[ref7] Roman M. G.; Houston R. Investigation of chloroplast regions rps16 and clpP for determination of *Cannabis sativa* crop type and biogeographical origin. Leg. Med. 2020, 47, 101759 10.1016/j.legalmed.2020.101759. 32711370

[ref8] Pavlovic R.; Nenna G.; Calvi L.; Panseri S.; Borgonovo G.; Giupponi L.; Cannazza G.; Giorgi A. Quality traits of “cannabidiol oils”: cannabinoids content, terpene fingerprint and oxidation stability of European commercially available preparations. Molecules 2018, 23, 1230 10.3390/molecules23051230. PMC6100014 29783790

[ref9] Hazekamp A.; Tejkalová K.; Papadimitriou S. Cannabis: from cultivar to chemovar II-a metabolomics approach to cannabis classification. Cannabis Cannabinoid Res. 2016, 1, 202–215. 10.1089/can.2016.0017.

[ref10] Fischedick J. T.; Glas R.; Hazekamp A.; Verpoorte R. A qualitative and quantitative HPTLC densitometry method for the analysis of cannabinoids in *Cannabis sativa* L.. Phytochem. Anal. 2009, 20, 421–426. 10.1002/pca.1143. 19609880

[ref11] Brighenti V.; Protti M.; Anceschi L.; Zanardi C.; Mercolini L.; Pellati F. Emerging challenges in the extraction, analysis and bioanalysis of cannabidiol and related compounds. J. Pharm. Biomed. Anal. 2021, 192, 113633 10.1016/j.jpba.2020.113633. 33039911

[ref12] Gülck T.; Møller B. L. Phytocannabinoids: origins and biosynthesis. Trends Plant Sci. 2020, 25, 985–1004. 10.1016/j.tplants.2020.05.005. 32646718

[ref13] Liu Y.; Victoria J.; Wood M.; Staretz M. E.; Brettell T. A. High performance thin-layer chromatography (HPTLC) analysis of cannabinoids in cannabis extracts. Forensic Chem. 2020, 19, 100249 10.1016/j.forc.2020.100249. PMC7352075 32676528

[ref14] Sanchez L.; Baltensperger D.; Kurouski D. Raman-based differentiation of hemp, cannabidiol-rich hemp, and cannabis. Anal. Chem. 2020, 92, 7733–7737. 10.1021/acs.analchem.0c00828. 32401504

[ref15] Sanchez L.; Filter C.; Baltensperger D.; Kurouski D. Confirmatory non-invasive and non-destructive differentiation between hemp and cannabis using a hand-held Raman spectrometer. RSC Adv. 2020, 10, 3212–3216. 10.1039/C9RA08225E. 35497720 PMC9048763

[ref16] del Mar Contreras M.; Jurado-Campos N.; Sánchez-Carnerero Callado C.; Arroyo-Manzanares N.; Fernández L.; Casano S.; Marco S.; Arce L.; Ferreiro-Vera C. Thermal desorption-ion mobility spectrometry: a rapid sensor for the detection of cannabinoids and discrimination of *Cannabis sativa* L. chemotypes. Sens. Actuators, B 2018, 273, 1413–1424. 10.1016/j.snb.2018.07.031.

[ref17] Bruni A.; Rodrigues C.; dos Santos C.; de Castro J.; Mariotto L.; Sinhorini L. Analytical Challenges for Identification of New Psychoactive Substances: A Literature-Based Study for Seized Drugs. Braz. J. Anal. Chem. 2021, 9, 52–78. 10.30744/brjac.2179-3425.RV-41-2021.

[ref18] Goutam S.; Goutam M.; Yadav P. Thin Layer Chromatographic Analysis of Psychoactive Plant *Cannabis sativa* L.. Int. J. Multidiscip. Approach Stud. 2015, 2, 166–175.

[ref19] Tsujikawa K.; Okada Y.; Segawa H.; Yamamuro T.; Kuwayama K.; Kanamori T.; Iwata Y. T. Thin-layer chromatography on silver nitrate-impregnated silica gel for analysis of homemade tetrahydrocannabinol mixtures. Forensic Toxicol. 2022, 40, 125–131. 10.1007/s11419-021-00592-9. 36454483

[ref20] Xu L.; Shu T.; Liu S. Simplified quantification of representative bioactives in food through TLC image analysis. Food Anal. Methods 2019, 12, 2886–2894. 10.1007/s12161-019-01645-x.

[ref21] van Beek T. A.; Subrtova D. Factors involved in the high pressure liquid chromatographic separation of alkenes by means of argentation chromatography on ion exchangers: overview of theory and new practical developments. Phytochem. Anal. 1995, 6, 1–19. 10.1002/pca.2800060102.

[ref22] Kaneti J.; de Smet L. C. P. M.; Boom R.; Zuilhof H.; Sudhölter E. J. R. Computational probes into the basis of silver ion chromatography. II. silver(I)–olefin complexes. J. Phys. Chem. A 2002, 106, 11197–11204. 10.1021/jp020994a.

[ref23] Damyanova B.; Momtchilova S.; Bakalova S.; Zuilhof H.; Christie W. W.; Kaneti J. Computational probes into the conceptual basis of silver ion chromatography: I. silver(I) ion complexes of unsaturated fatty acids and esters. J. Mol. Struct.: THEOCHEM 2002, 589–590, 239–249. 10.1016/S0166-1280(02)00281-6.

[ref24] Huang S.; Claassen F. W.; van Beek T. A.; Chen B.; Zeng J.; Zuilhof H.; Salentijn G. I. J. Rapid distinction and semiquantitative analysis of THC and CBD by silver-impregnated paper spray mass spectrometry. Anal. Chem. 2021, 93, 3794–3802. 10.1021/acs.analchem.0c04270. 33576613 PMC8023514

[ref25] Zhou B.; Tan M.; Lu J.; Zhao J.; Xie A.; Li S. Simultaneous determination of five active compounds in *Chimonanthus nitens* by double-development HPTLC and scanning densitometry. Chem. Cent. J. 2012, 6, 46 10.1186/1752-153X-6-46. 22616568 PMC3414808

[ref26] Stroka J.; Anklam E. Development of a simplified densitometer for the determination of aflatoxins by thin-layer chromatography. J. Chromatogr. A 2000, 904, 263–268. 10.1016/S0021-9673(00)00947-X. 11204240

[ref27] Chen W.; Yao Y.; Chen T.; Shen W.; Tang S.; Lee H. K. Application of smartphone-based spectroscopy to biosample analysis: a review. Biosens. Bioelectron. 2021, 172, 112788 10.1016/j.bios.2020.112788. 33157407

[ref28] Fan Y.; Li J.; Guo Y.; Xie L.; Zhang G. Digital image colorimetry on smartphone for chemical analysis: a review. Measurement 2021, 171, 108829 10.1016/j.measurement.2020.108829.

[ref29] Ross G. M. S.; Bremer M. G. E. G.; Nielen M. W. F. Consumer-friendly food allergen detection: moving towards smartphone-based immunoassays. Anal. Bioanal. Chem. 2018, 410, 5353–5371. 10.1007/s00216-018-0989-7. 29582120 PMC6096701

[ref30] Silver J. Let us teach proper thin layer chromatography technique!. J. Chem. Educ. 2020, 97, 4217–4219. 10.1021/acs.jchemed.0c00437.

[ref31] Sarma N. D.; Waye A.; ElSohly M. A.; Brown P. N.; Elzinga S.; Johnson H. E.; Marles R. J.; Melanson J. E.; Russo E.; Deyton L.; Hudalla C.; Vrdoljak G. A.; Wurzer J. H.; Khan I. A.; Kim N.-C.; Giancaspro G. I. Cannabis inflorescence for medical purposes: USP considerations for quality attributes. J. Nat. Prod. 2020, 83, 1334–1351. 10.1021/acs.jnatprod.9b01200. 32281793

[ref32] Tsumura Y.; Aoki R.; Tokieda Y.; Akutsu M.; Kawase Y.; Kataoka T.; Takagi T.; Mizuno T.; Fukada M.; Fujii H.; Kurahashi K. A survey of the potency of Japanese illicit cannabis in fiscal year 2010. Forensic Sci. Int. 2012, 221, 77–83. 10.1016/j.forsciint.2012.04.005. 22554871

[ref33] França H. S.; Acosta A.; Jamal A.; Romao W.; Mulloor J.; Almirall J. R. Experimental and ab initio investigation of the products of reaction from Δ9-tetrahydrocannabinol (Δ9-THC) and the fast blue BB spot reagent in presumptive drug tests for cannabinoids. Forensic Chem. 2020, 17, 100212 10.1016/j.forc.2019.100212.

[ref34] Galand N.; Ernouf D.; Montigny F.; Dollet J.; Pothier J. Separation and identification of cannabis components by different planar chromatography techniques (TLC, AMD, OPLC). J. Chromatogr. Sci. 2004, 42, 130–134. 10.1093/chromsci/42.3.130. 15023248

[ref35] Momchilova S.; Nikolova-Damyanova B. Stationary phases for silver ion chromatography of lipids: preparation and properties. J. Sep. Sci. 2003, 26, 261–270. 10.1002/jssc.200390032.

[ref36] McRae G.; Melanson J. E. Quantitative determination and validation of 17 cannabinoids in cannabis and hemp using liquid chromatography-tandem mass spectrometry. Anal. Bioanal. Chem. 2020, 412, 7381–7393. 10.1007/s00216-020-02862-8. 32833075 PMC7533253

[ref37] Scheunemann A.; Elsner K.; Germerott T.; Hess C.; Röhrich J. Simultaneous quantification of 18 different phytocannabinoids in serum using a highly sensitive liquid chromatography-tandem mass spectrometry (LC-MS/MS) method. J. Chromatogr. B 2021, 1173, 122685 10.1016/j.jchromb.2021.122685. 33882447

[ref38] Gul W.; Gul S. W.; Radwan M. M.; Wanas A. S.; Mehmedic Z.; Khan I. I.; Sharaf M. H. M.; ElSohly M. A. Determination of 11 Cannabinoids in Biomass and Extracts of Different Varieties of Cannabis Using High-Performance Liquid Chromatography. J. AOAC Int. 2015, 98, 1523–1528. 10.5740/jaoacint.15-095. 26651563

[ref39] Köguel C. C.; López-Pelayo H.; Balcells-Olivero M. M.; Colom J.; Gual A. Psychoactive constituents of cannabis and their clinical implications: a systematic review. Adicciones 2018, 30, 140–151. 10.20882/adicciones.858. 28492950

[ref40] Gaoni Y.; Mechoulam R. Isolation and structure of Δ^1^-tetrahydrocannabinol and other neutral cannabinoids from hashish. J. Am. Chem. Soc. 1971, 93, 217–224. 10.1021/ja00730a036. 5538858

[ref41] Jang E.; Kim H.; Jang S.; Lee J.; Baeck S.; In S.; Kim E.; Kim Y.-u.; Han E. Concentrations of THC, CBD, and CBN in commercial hemp seeds and hempseed oil sold in Korea. Forensic Sci. Int. 2020, 306, 110064 10.1016/j.forsciint.2019.110064. 31786513

[ref42] Hazekamp A. The Trouble with CBD Oil. Med. Cannabis Cannabinoids 2018, 1, 65–72. 10.1159/000489287. 34676324 PMC8489347

[ref43] Liebling J. P.; Clarkson N. J.; Gibbs B. W.; Yates A. S.; O’Sullivan S. E. An analysis of over-the-counter cannabidiol products in the United Kingdom. Cannabis Cannabinoid Res. 2022, 7, 207–213. 10.1089/can.2019.0078. 33998849 PMC9070743

