# Supplementary material for: Semiquantitative Screening of THC Analogues by Silica Gel TLC with an Ag(I) Retention Zone and Chromogenic Smartphone Detection
Source: Anal Chem. 2022 Sep 30;94(40):13710–8. doi: 10.1021/acs.analchem.2c01627 (PMC9558087; doi:10.1021/acs.analchem.2c01627)
Supplement: Supplementary file 1 — ac2c01627_si_001.pdf [file ac2c01627_si_001.pdf]

Supporting Information

Semi-quantitative screening of THC analogs by silica gel TLC with an Ag(I) retention zone and chromogenic smartphone detection

Si Huang,[a,b] Ruiying Qiu,[a] Zhengfa Fang,[a] Ke Min,[a] Teris A. van Beek,[b] Ming Ma,[a] Bo Chen,[a*] Han Zuilhof,[a,b,d,*] and Gert IJ. Salentijn[b,c*]

[a]Key Laboratory of Phytochemical R&D of Hunan Province and Key Laboratory of Chemical Biology & Traditional Chinese Medicine Research of Ministry of Education, Hunan Normal University, Changsha 410081, China.

[b]Laboratory of Organic Chemistry, Wageningen University, Wageningen 6708 WE, The Netherlands.

[c]Wageningen Food Safety Research (WFSR), Wageningen University & Research, Wageningen 6700 AE, The Netherlands.

[d]Department of Chemical and Materials Engineering, Faculty of Engineering, King Abdulaziz University, Jeddah 21589, Saudi Arabia.

E-mail corresponding authors:

dr-chenpo@vip.sina.com; Han.Zuilhof@wur.nl; Gert.Salentijn@wur.nl

Table of Contents

Figure S1	HPLC-UV calibration curves of THC, CBN, THCA, CBD, CBG and CBDA	Page S3
Table S1	THCA, CBN, THC, CBD, CBG and CBDA content in samples	Page S4
Table S2	Preparation of mixed cannabis extracts	Page S5
Figure S2	Design of Ag(I)-TLC plate	Page S6
Protocol S1	Optimization of TLC plates	Page S7
Figure S3	Images of different storage conditions for Ag(I)-TLC plates	Page S8
Figure S4	Glass spray bottle for spraying color reagents	Page S8
Figure S5	Images of light box for controlling photographing conditions	Page S9
Figure S6	Photographs of Ag(I)-TLC plates for parameter optimization	Page S10
Figure S7	Resolution of standards and analysis time under different experimental parameters by Ag(I)-TLC	Page S11
Protocol S2	Procedure for extracting cannabinoids from TLC plates	Page S13
Table S3	LC-MS/MS acquisitions parameters for the 12 cannabinoids	Page S14
Figure S8	Image of separation of THC and CBD on Ag(I)-TLC plates stored under different conditions and duration	Page S15
Figure S9	Absolute R_f of THC and CBD obtained by Ag (I)-TLC plates stored under different conditions and duration	Page S16
Figure S10	Photographs of preparative normal TLC and Ag(I)-TLC for separation of ‘standard_mixture’ and ‘sample_mixture’	Page S17
Figure S11	HPLC-MRM chromatograms of cannabinoids in ‘standard_mixture’ before and after separation by normal TLC and Ag(I)-TLC	Page S18
Figure S12	HPLC-MRM chromatograms of cannabinoids in ‘sample_mixture’ before and after separating by normal TLC and Ag(I)-TLC	Page S19
Figure S13	Chemical structures of cannabinoids separated by Ag(I)-TLC plate	Page S20
Figure S14	Calibration curves between THC, CBN, THCA amount on Ag(I)-TLC plate and smartphone signal	Page S20
Figure S15	Images of various amounts of THC, CBN and THCA analyzed by Ag(I)-TLC plate	Page S20
Figure S16	Photographs of Ag(I)-TLC plates with THC+CBN standard mixtures and relationship between normalized saturation signal and CBN/(THC+CBN) ratio	Page S21
Figure S17	Relationship between normalized (B−R) signal and CBN/(THC+CBN) ratio	Page S23
Figure S18	Images of samples accompanied with standards analyzed by Ag(I)-TLC plate	Page S24
Figure S19	Images of samples analyzed by Ag(I)-TLC plate	Page S25
Figure S20	Images of various amounts of CBD, CBG and CBDA analyzed by Ag(I)-TLC plate	Page S27
References		Page S28

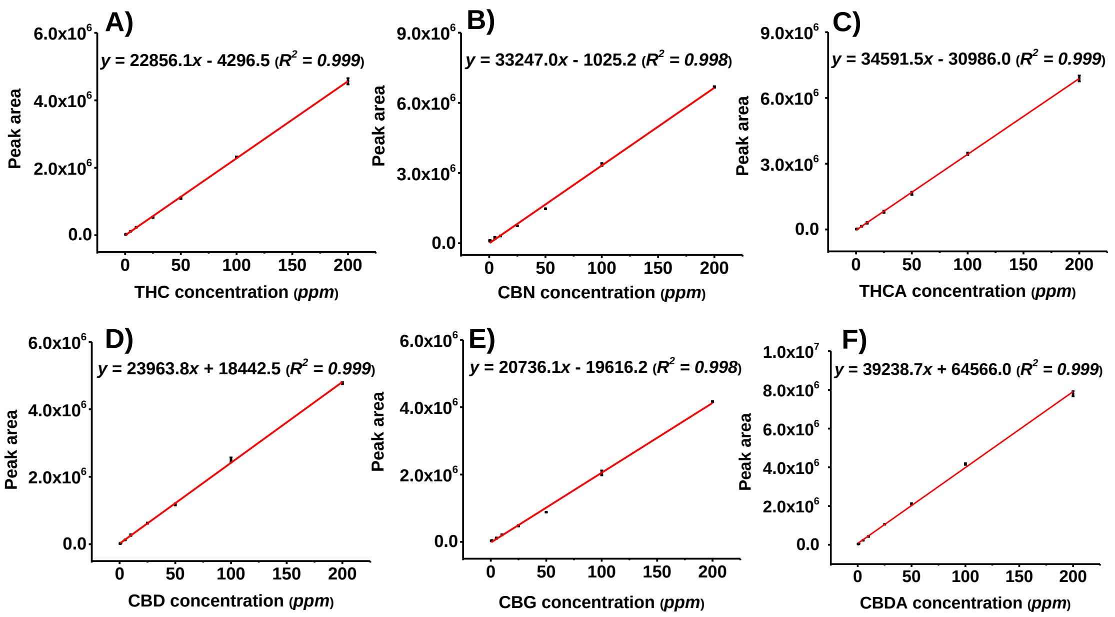

Figure S1. Calibration curves between peak area and concentration of (A) THC, (B) CBN, (C) THCA, (D) CBD, (E) CBG, and (F) CBDA constructed by HPLC-UV. Error bars represent the standard deviation (n=3).

1.00 mg·mL^{-1} of THCA, THC, CBN, CBD, CBG and CBDA methanol stock solutions were diluted with methanol to obtain eight calibration solutions for each compound with concentrations of 0.50, 1.00, 5.00, 10.00, 25.00, 50.00, 100.00, 200.00 μg·mL^{-1}. 10 μL of each the above solutions was injected for HPLC-UV analysis with UV detection at 228 nm. The constructed calibration curves were used as external standard to quantify the concentrations of these six cannabinoids in sample extracts.

Table S1. Contents of THCA, CBN, THC, CBD, CBG and CBDA in samples.

LC-UV analysis of Sample extract	THCA	CBN	CBD	THC	CBG	CBDA
Cannabis_1	0.216% (4.0%)	0.100% (1.8%)	0.028% (8.4%)	0.047% (5.1%)	ND	ND
Cannabis_2	0.052% (1.4%)	0.070% (1.1%)	ND	0.010% (1.6%)	ND	ND
Cannabis_3	ND	ND	ND	ND	ND	ND
1.00 $mg \cdot mL^{-1}$ Marijuana extract concentrate	44.2 $\mu g \cdot mL^{-1}$ (2.8%)	62.2 $\mu g \cdot mL^{-1}$ (4.6%)	ND	146.8 $\mu g \cdot mL^{-1}$ (3.8%)	9.9 $\mu g \cdot mL^{-1}$ (5.3%)	ND
CBD oil	ND	ND	104.2 $mg \cdot mL^{-1}$ (5.1%)	ND	ND	ND

ND means not detected.

Values in parentheses represent relative standard deviations (n=3).

Sample preparation procedure was described in the main manuscript. Concentrations of the six cannabinoids were calculated by the calibration curves from Figure S1. Subsequently, cannabinoid percentage was expressed as follows:

%THCA=[THCA]×(VOL/DW) ×100

%THC, %CBN, CBD%, CBG% and CBDA% were calculated similarly;

[THCA]: concentration of THCA ($\mu g \cdot mL^{-1}$), VOL=sample volume (0.3 mL), DW=dry sample weight (100.0 mg).

Table S2. The preparation of mixed cannabis extracts set I and II.

mixed cannabis extracts set	Volume of 10.00 mg·mL^{-1} marijuana solution	cannabis_3 leaf	CBD powder	MeOH
I	0.0–0.6 mL*	100.0 mg	-	0.3 mL
II	0.0–0.6 mL*	100.0 mg	1.00 mg	0.3 mL

*This range includes 0, 0.12, 0.14, 0.2, 0.3, 0.4, 0.5, 0.6 mL.

Specifically, 0, 0.12, 0.14, 0.2, 0.3, 0.4, 0.5, 0.6 mL of 10.00 mg·mL^{-1} marijuana solution were individually transferred to 2.0 ml brown autosampler vials, and then dried under a flow of nitrogen. Afterwards, to each vial 100.0 mg of cannabis_3 leaf (set I) or 1.00 mg of CBD powder and 100.0 mg of cannabis_3 leaf (set II) were added. Subsequently, 300 μL of MeOH was added to each vial and then the mixtures were vortexed vigorously during 3 min.

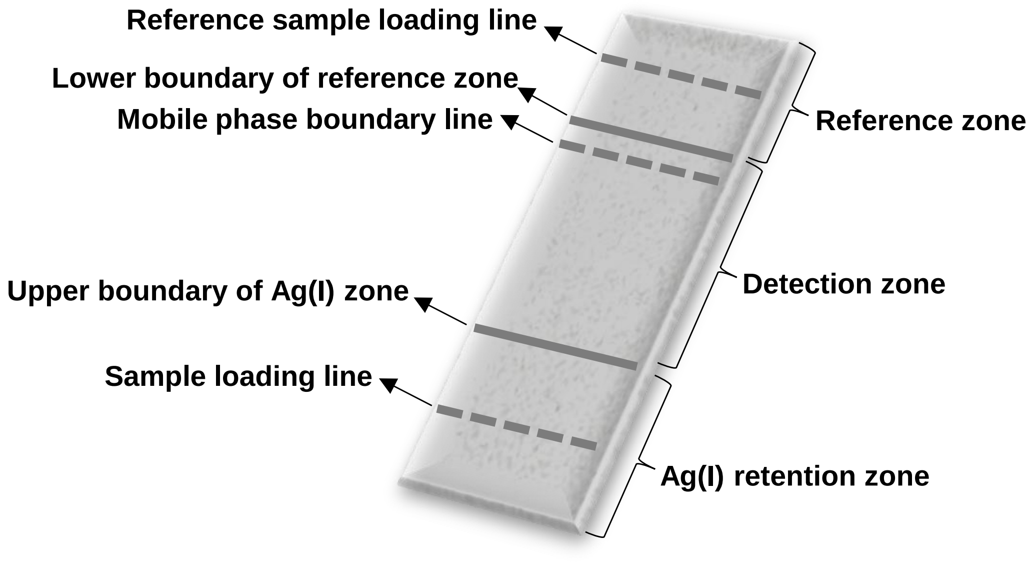

Figure S2. Design of Ag(I)-TLC plate. (Same as part of Figure 2 in manuscript)

The TLC plate was divided into three zones, from bottom to top: (1) Ag(I) retention zone, (2) separation and detection zone and (3) reference zone, by drawing two solid lines with a 2B pencil. The lower solid line represents the upper boundary of the Ag(I) zone. The upper solid line represents the lower boundary of the reference zone which is 10 mm removed from the top of the TLC plate. In the Ag(I) retention zone, there is a sample loading line, which is 10 mm removed from the bottom of the plate. In the separation and detection zone, there is a mobile phase boundary line which is 12 mm removed from the top of the plate. In the reference zone, there is a reference sample loading line which is in the middle of this zone, namely 5 mm below the top of the plate. When performing TLC parameter optimization experiments, there was no reference zone so that the TLC plate was 1 cm shorter than the above-described TLC plate. TLC plates were immersed in a $AgNO_3$ solution to approximately 1 mm below the upper boundary of the Ag(I) zone for around 3 seconds and then taken out in dimmed light. The $AgNO_3$ solution would wet another 1 mm to reach the boundary by capillary force. Hereafter, the modified TLC plates were placed in the fume hood with the light off for air drying or blow drying with a hair dryer. The dry plates (named “Ag(I)-TLC” plates) were immediately used or stored in plastic sealed bags with the aluminum side up.

Protocol S1. Optimization of TLC plates.

The total height of the TLC plate was first optimized by using TLC plates with a height of 4, 5, 6, 7 and 8 cm. The Ag(I) retention zone was 18 mm and the $AgNO_3$ solution for modification was 2.00 M. 1.0 μL of 1.00 μg·μL^{-1} THCA, THC, CBN, CBDA, CBD and CBG solutions was loaded on the sample loading line of the TLC plate with a pipette (Eppendorf 3120000216, 0.1–2.5 μL). After drying, the TLC plate was developed with the mobile phase and sprayed for color development. Developing time of each TLC plate as well as developed height and diameters of each spot were recorded. Resolution of spots was calculated according to the equation: $\frac{H2-H1}{0.5\times(D2+D1)}$ (H2 and H1 represent the developed heights of two spots; D2 and D1 represent the diameters of two spots). Subsequently, the optimized TLC total height was chosen for optimization of $AgNO_3$ concentrations. The height of the Ag(I) retention zone was kept at 18 mm. Solutions of 0.90, 1.20, 1.50, 1.80 or 2.00 M of $AgNO_3$ in ACN were prepared in brown bottles for modification of the Ag(I) retention zone. Next, the separation performance was compared according to the same procedure as for TLC total height optimization. Next, TLC plates with optimized total height and $AgNO_3$ concentration with Ag(I) retention zone heights of 14, 17, 20, 23 or 26 mm were evaluated following the above-mentioned steps. Finally, optimized TLC plates were dried either by air or blow dryer after Ag(I) modification. Each optimization experiment was repeated three times.

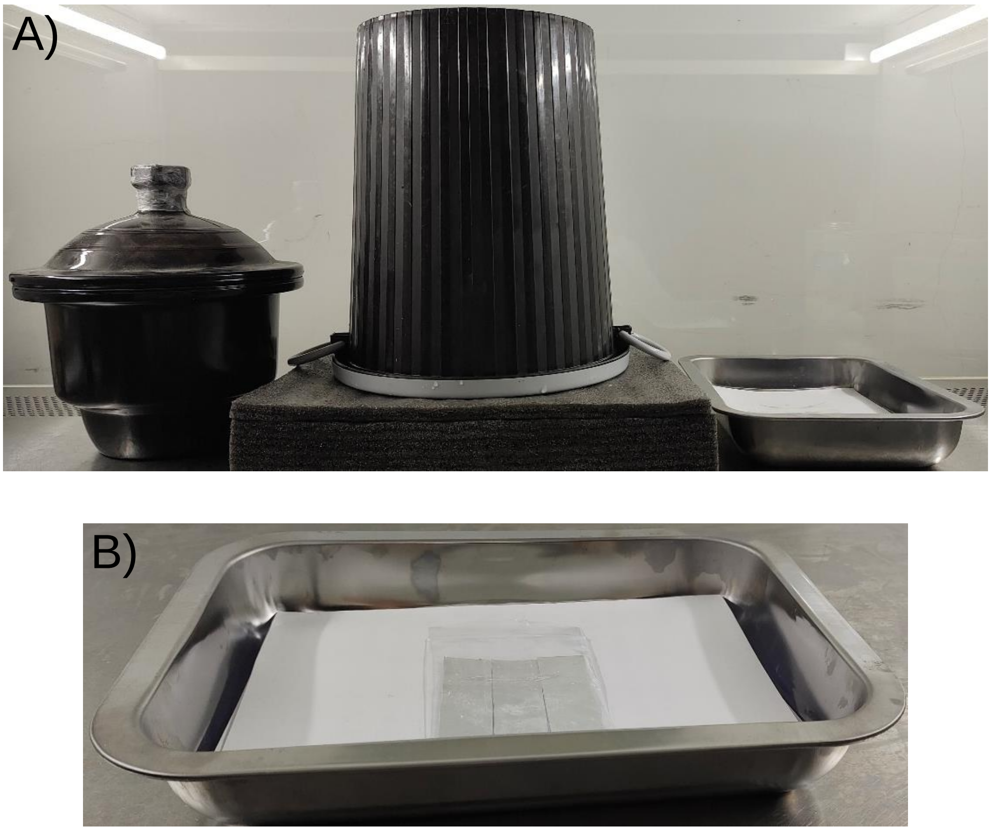

Figure S3. Image of (A) three different storage conditions, and (B) Ag(I)-TLC plates in plastic sealed bag with aluminum side up.

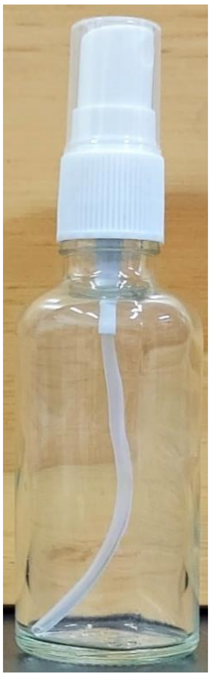

Figure S4. Glass spray bottle used for spraying color reagents.

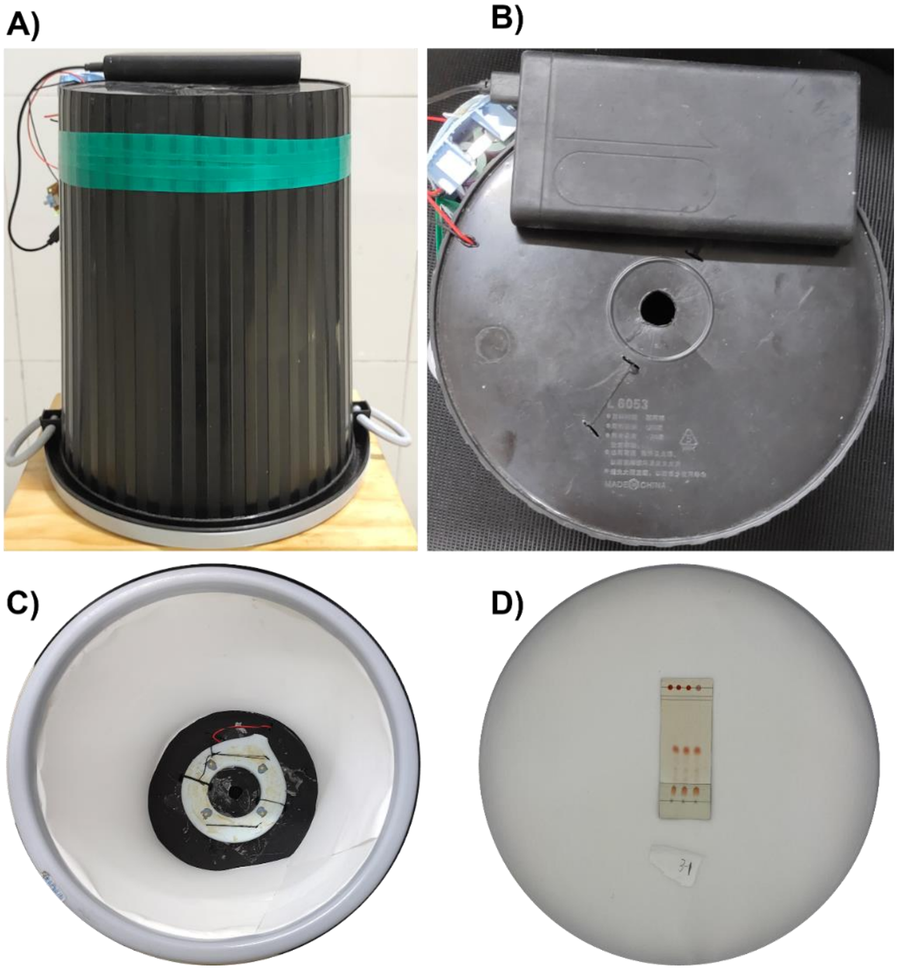

Figure S5. Images of (A) custom-made light box, (B) top window for smartphone camera, (C) light source, and (D) example of acquired image.

The light box is a modified cylindrical trash can, with a height of 28 cm, a top diameter of 20 cm, and a bottom diameter of 25 cm. A small hole with a diameter of 1.5 cm is in the middle of the top for taking photos with a smartphone. The inner wall of the light box is covered with white rough filter paper to scatter the light irradiated to the surroundings and prevent the light from being reflected to the TLC plate to avoid background interference of different intensities. The light source consists of four small LED lights in a uniform ring shape with a total power of 1.2 W. The light source is controlled by its own switch and powered by a mobile power bank with a capacity of 10 Ah.

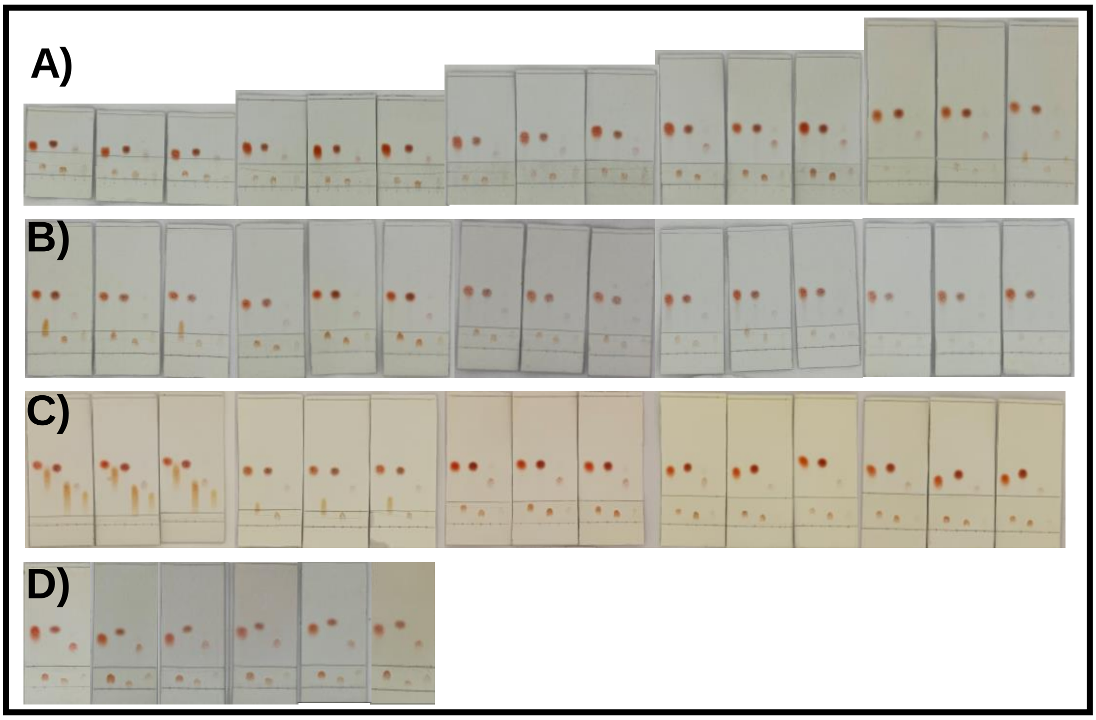

Figure S6. Images of parameter optimization experiments including (A) height of TLC plate, (B) concentration of $AgNO_3$, (C) height of Ag (I) zone, and (D) drying methods of modified plates. Standards loaded on the plates from left to right were 1.0 μL of 1.00 μg·μL^{-1} THC, CBD, CBN, CBG, THCA and CBDA. Three adjacent TLC plates represent three repeat experiments.

To obtain the best possible performance of Ag(I)-TLC plates, a series of parameters were optimized, including the height of Ag(I) retention zone, the total height of the TLC plate, the concentration of $AgNO_3$ solution for modification and the drying methods for Ag(I)-TLC plates (images of the plates are shown in Figure S6). The resolution between the lowest spot of the THC analogs and the highest spot of the CBD analogs was recorded to evaluate the separation performance. When using an Ag(I)-TLC plate, THCA has the lowest R_f of the three THC analogs (THC, CBN and THCA) and CBD has the highest R_f of the three CBD analogs (CBD, CBG and CBDA). Additionally, the resolution between THC and THCA as well as THC and CBN were also taken into account to evaluate the separation of the THC analogs. Since THC and CBN had very similar R_f values in all optimization experiments, resolution between CBN and THCA was approximately the same as the resolution between THC and THCA. The time required for development of the TLC plates under different conditions was recorded as well.

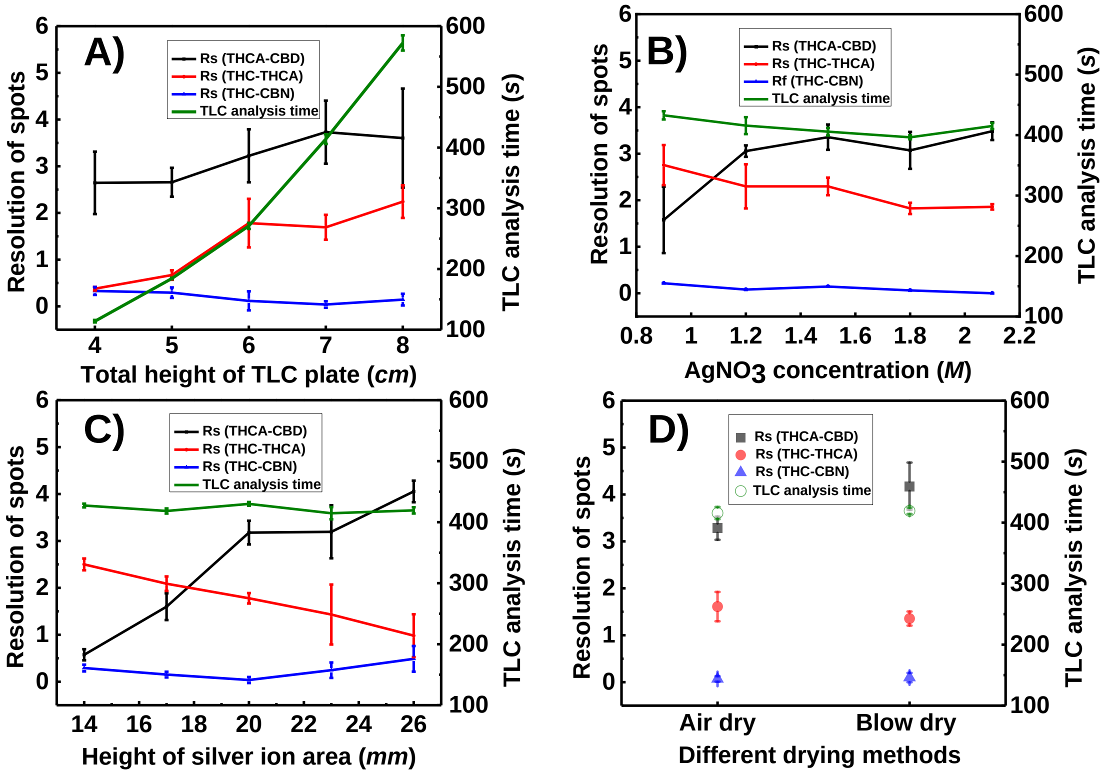

Figure S7. THC, CBD, CBN, CBG, THCA and CBDA standards analyzed on an Ag(I)-TLC plate with various (A) total height of TLC plate, (B) concentration of $AgNO_3$, (C) length of Ag(I) retention zone and (D) drying methods. Error bars represent the standard deviation (n=3).

Optimization of total height of the TLC plate

As shown in Figure S7A, with the increasing total height of the TLC plate, the analysis time increased, while the resolution of THCA-CBD first increased and remained constant above a total height of 7 cm. The resolution of THC-THCA increased a lot from a TLC height of 4 cm to 6 cm, and then remained constant at a height of 6 cm and 7 cm. When the total height was further increased, greater resolution of THC-THCA could be achieved but the CBD spot tended to migrate out of the Ag(I) retention zone due to the longer running time. Therefore, a total TLC height of 7 cm was chosen for all further analyses.

Optimization of $AgNO_3$ solution concentration for modification

Next, the influence of the $AgNO_3$ concentration on the resolution and analysis time was investigated (Figure S7B). The analysis time was almost not influenced by the $AgNO_3$

concentration. The best resolution for both THCA-CBD and THC-THCA was achieved when the $AgNO_3$ concentration was 1.5 M.

Optimization of height of Ag(I) retention zone

With the above parameters fixed, the height of the Ag(I) retention zone was varied from 14 mm to 26 mm and its effect on the resolution observed (Figure S7C). The separation of the pair THCA-CBD improved strongly from 14-20 mm and less so from 20-26 mm. The resolution of THC-THCA decreased with an increasing height of the Ag(I) zone. Even worse, if the Ag(I) retention zone was too long, THC analogs were retained in the Ag(I) zone and this interfered with the color analysis. Therefore, a height of 20 mm was selected for the Ag(I) retention zone.

Optimization of drying methods for Ag(I)-TLC plates

The $AgNO_3$ for the modification was dissolved in acetonitrile. Passive drying and accelerated drying with a blow dryer were compared for the separation performance of three pairs of standards (Figure S7D). Accelerated drying leads to better resolution of both THCA-CBD and THC-THCA. This is probably because accelerated drying leads to a more complete evaporation of acetonitrile (boiling point 82 °C) and thus avoids competition of ACN and cannabinoids for binding with Ag(I) considering that acetonitrile can complex with Ag(I).[2]

Based on the above, the optimized TLC plate is 7 cm high, has a 20 mm-high Ag(I) retention zone modified with 1.5 M $AgNO_3$ solution and dried by blow dryer. All the above optimized parameters were adopted in later experiments.

Protocol S2. Procedure for extracting cannabinoids from TLC plates.

20.0 μL of the 'standard_mixture' and 'sample_mixture' were loaded on four Ag(I)-TLC plates with a width of 9 cm and four unmodified TLC plates, otherwise identical in design. After development, one Ag(I)-TLC plate for 'standard_mixture' and 'sample_mixture' as well as one unmodified TLC plate for 'standard_mixture' and 'sample_mixture' were sprayed with the color reagents. For the remaining Ag(I)-TLC plates, each plate was divided into two parts (upper part and lower part) along the upper boundary of Ag (I) zone by scissors. The silica gel in the upper part and lower part of each plate was scraped off separately and extracted with 1.0 ml MeOH by vortexing for 3 min. The supernatant was removed and another 1.0 mL fresh MeOH was added to the silica for vortexing for another 3 min. Subsequently, supernatants from both extractions were combined and filtered using 0.2 μm PTFE membrane syringe filters. After evaporation of the solvent with nitrogen and reconstitution in 200 μL of MeOH, all extracts were analyzed by HPLC-MS/MS with the MRM transitions shown in Table S3.

Table S3. LC-MS/MS acquisitions parameters for the 12 cannabinoids.[1]

Name	Q1 (*m/z*)	Q3 (*m/z*)	Q3 (*m/z*)	CE1 (eV)	CE2 (eV)	Dwell (ms)	RT (min)
CBDV	287.2	165.1	123.1	23	30	40	3.83
THCV	287.2	165.1	123.1	23	30	40	5.94
CBD	315.2	193.1	135.1	21	20	40	5.15
THC	315.2	193.1	135.1	21	20	40	9.28
CBG	317.2	193.1	123.1	16	32	40	4.89
CBN	311.2	223.1	241.1	22	18	40	7.62
CBDVA	313.2	191.1	233.1	26	20	40	3.59
THCVA	313.2	191.1	233.1	26	20	40	7.62
CBDA	359.2	219.1	261.1	32	25	40	4.66
THCA	359.2	219.1	261.1	32	25	40	12.47
CBLA	359.2	219.1	261.1	32	25	40	13.96
CBGA	343.2	219.1	261.1	23	16	40	4.89

CBDV: cannabidivarin; THCV: tetrahydrocannabivarin; CBD: cannabidiol; THC: Δ9-tetrahydrocannabinol; CBG: cannabigerol; CBN: cannabinol; CBDVA: cannabidivarinic acid; THCVA: tetrahydrocannabivarin carboxylic acid; CBDA: cannabidiolic acid; THCA: tetrahydrocannabinolic acid; CBLA: cannabicyclolic acid; CBGA: cannabigerolic acid.

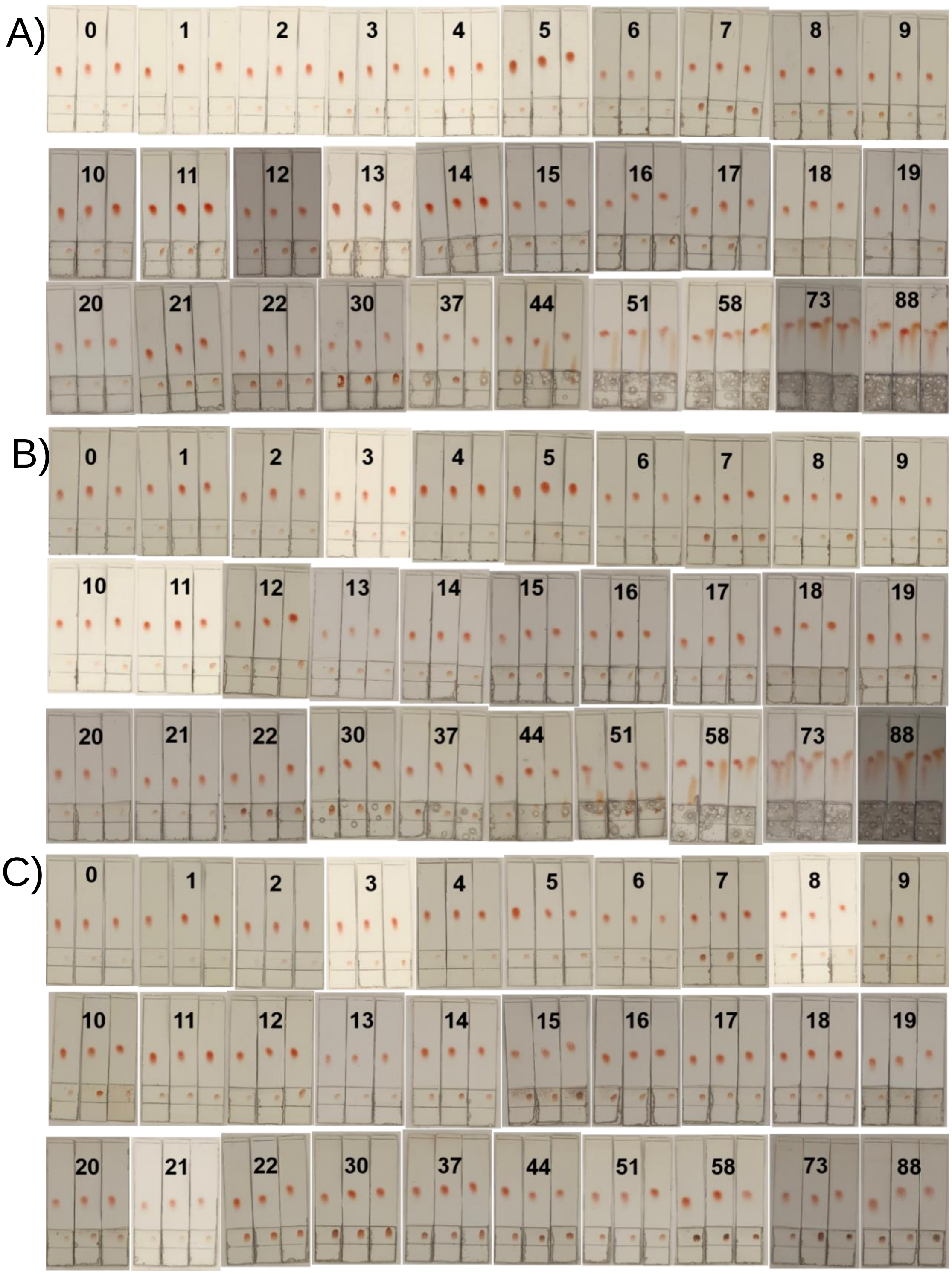

Figure S8. Images of separation performance obtained by Ag(I)-TLC plates stored (A) on the desk, (B) in the black box, and (C) in the desiccator. The left spot on each TLC is for THC and the right spot for CBD.

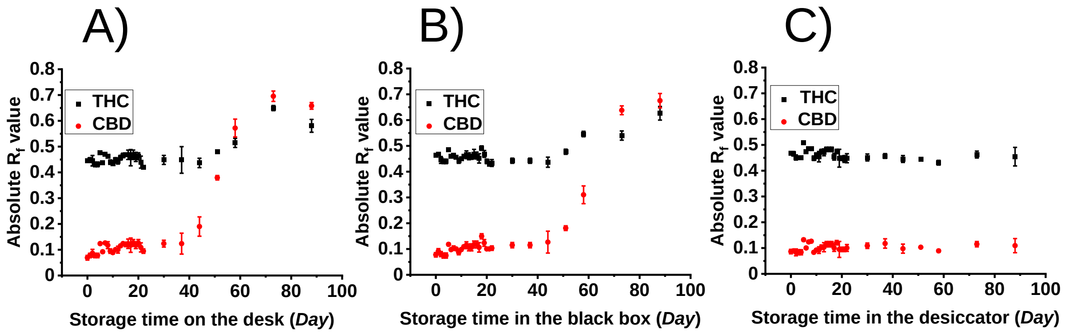

Figure S9. Absolute R_f of THC and CBD obtained by Ag(I)-TLC plates stored (A) on the desk, (B) in the black box, and (C) in the desiccator. Error bars represent the standard deviation (n=3).

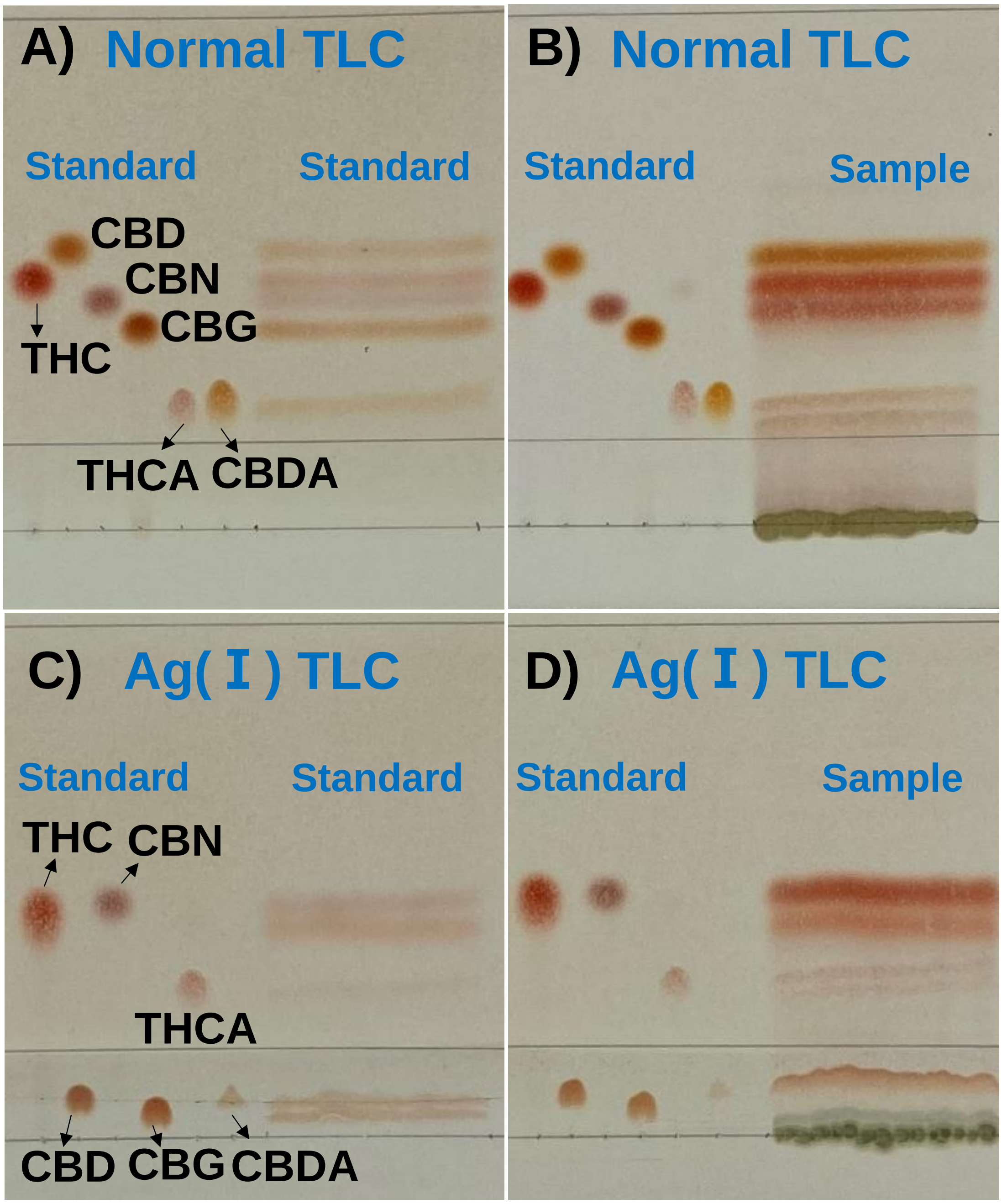

Figure S10. Standard (A, C) and sample (B, D) mixture analyzed by (A, B) unmodified TLC and (C, D) Ag(I)-TLC plate.

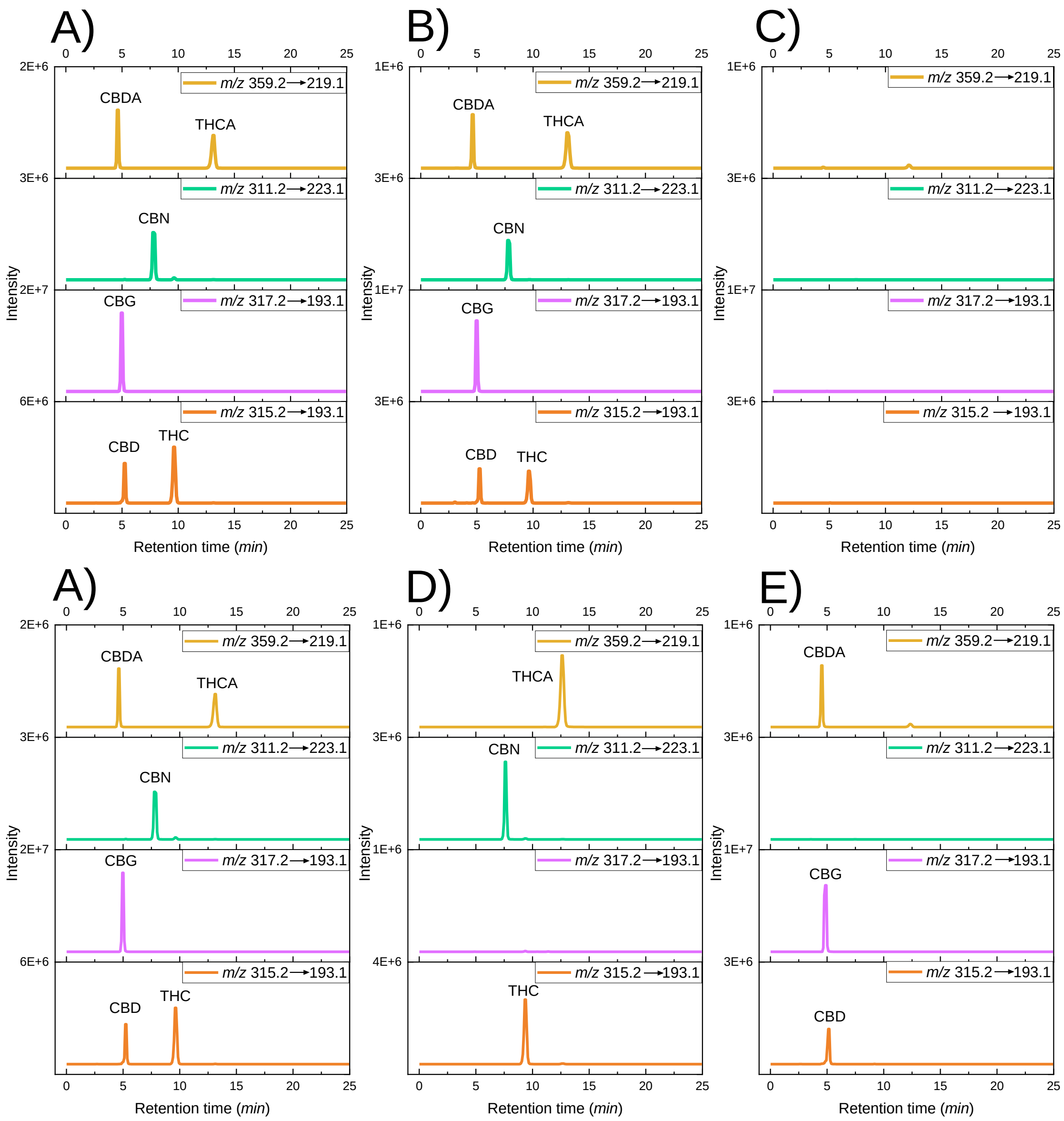

Figure S11. HPLC-MRM chromatograms of cannabinoids in (A) ‘standard_mixture’, (B) extract of normal TLC upper part silica after separating ‘standard_mixture’, (C) extract of normal TLC lower part silica after separating ‘standard_mixture’, (D) extract of Ag(I)-TLC detection zone silica after separating ‘standard_mixture’, (E) extract of Ag (I) retention zone silica after separating ‘standard_mixture’. The data in A, D and E are identical to Figure 5 in the main text, and presented for convenient comparison

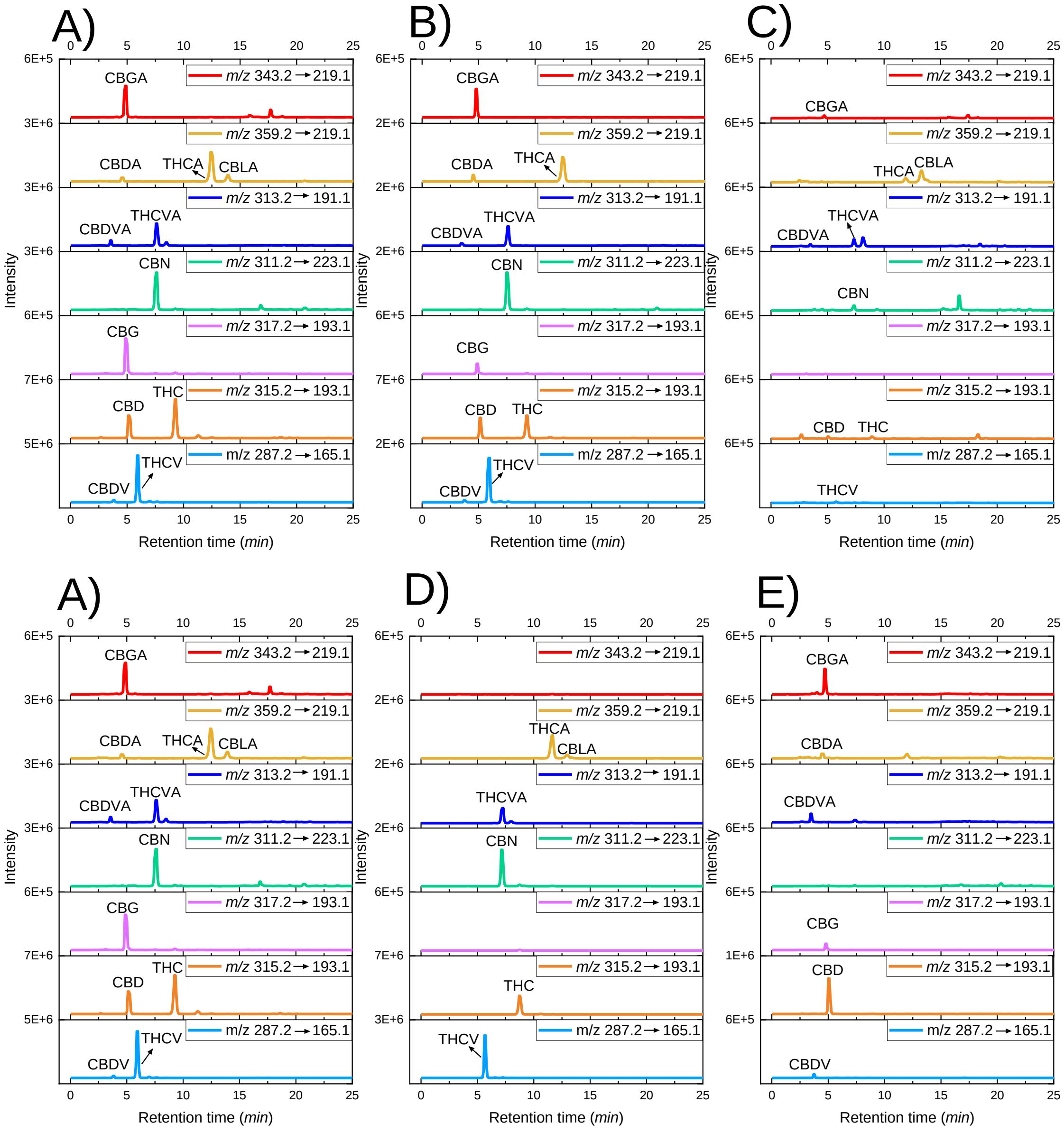

Figure S12. HPLC-MRM chromatograms of cannabinoids in (A) ‘sample_mixture’, (B) extract of normal TLC upper part silica after separating ‘sample_mixture’, (C) extract of normal TLC lower part silica after separating ‘sample_mixture’, (D) extract of Ag(I)-TLC detection zone silica after separating ‘sample_mixture’, (E) extract of Ag (I) retention zone silica after separating ‘sample_mixture’. A is presented twice for convenient comparison.

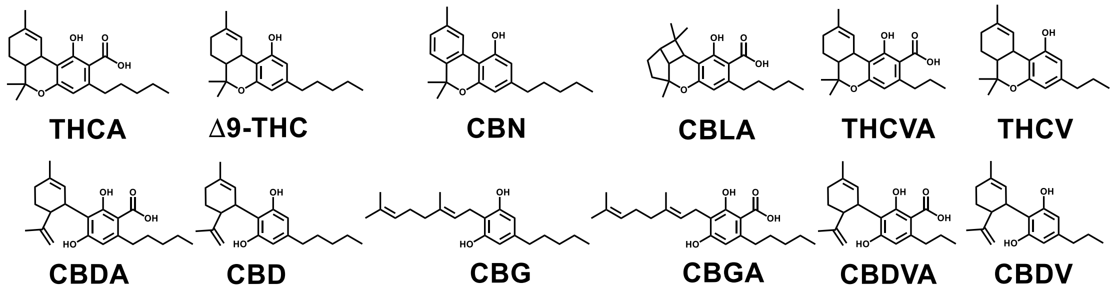

Figure S13. Detected cannabinoids separated by Ag (I)-TLC plate.

(CBDV: cannabidivarin; THCV: tetrahydrocannabivarin; CBD: cannabidiol; THC: Δ9-tetrahydrocannabinol; CBG: cannabigerol; CBN: cannabinol; CBDVA: cannabidivarinic acid; THCVA: tetrahydrocannabivarin carboxylic acid; CBDA: cannabidiolic acid; THCA: tetrahydrocannabinolic acid; CBLA: cannabicyclolic acid; CBGA: cannabigerolic acid.)

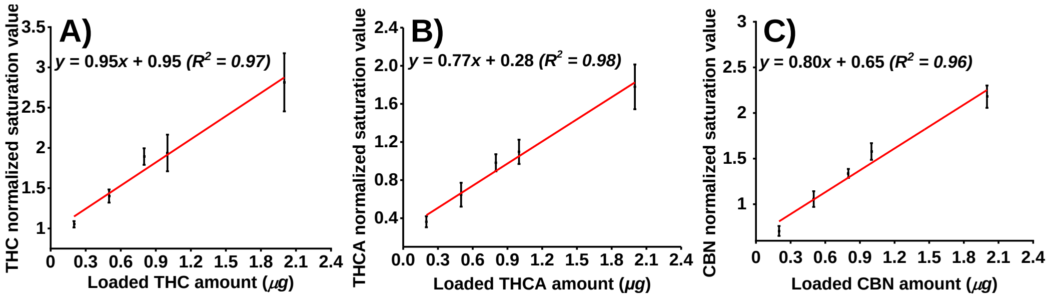

Figure S14. Calibration curves between loaded (A) THC, (B) THCA or (C) CBN and saturation. Error bars represent the standard deviation (n=3).

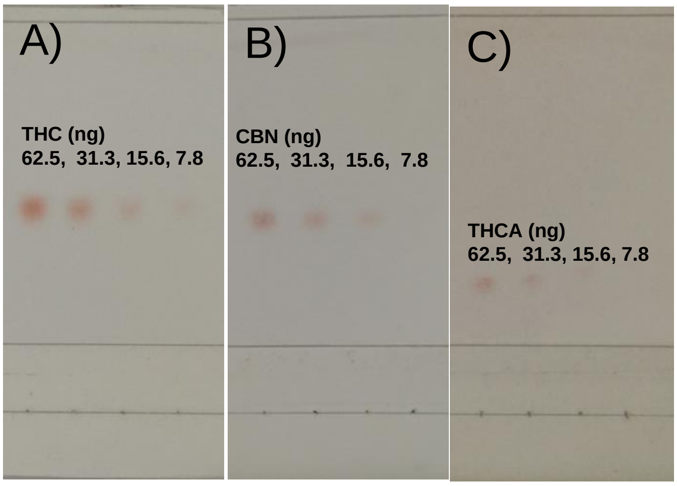

Figure S15. Various amounts of (A) THC, (B) CBN and (C) THCA standards analyzed by Ag(I)-TLC.

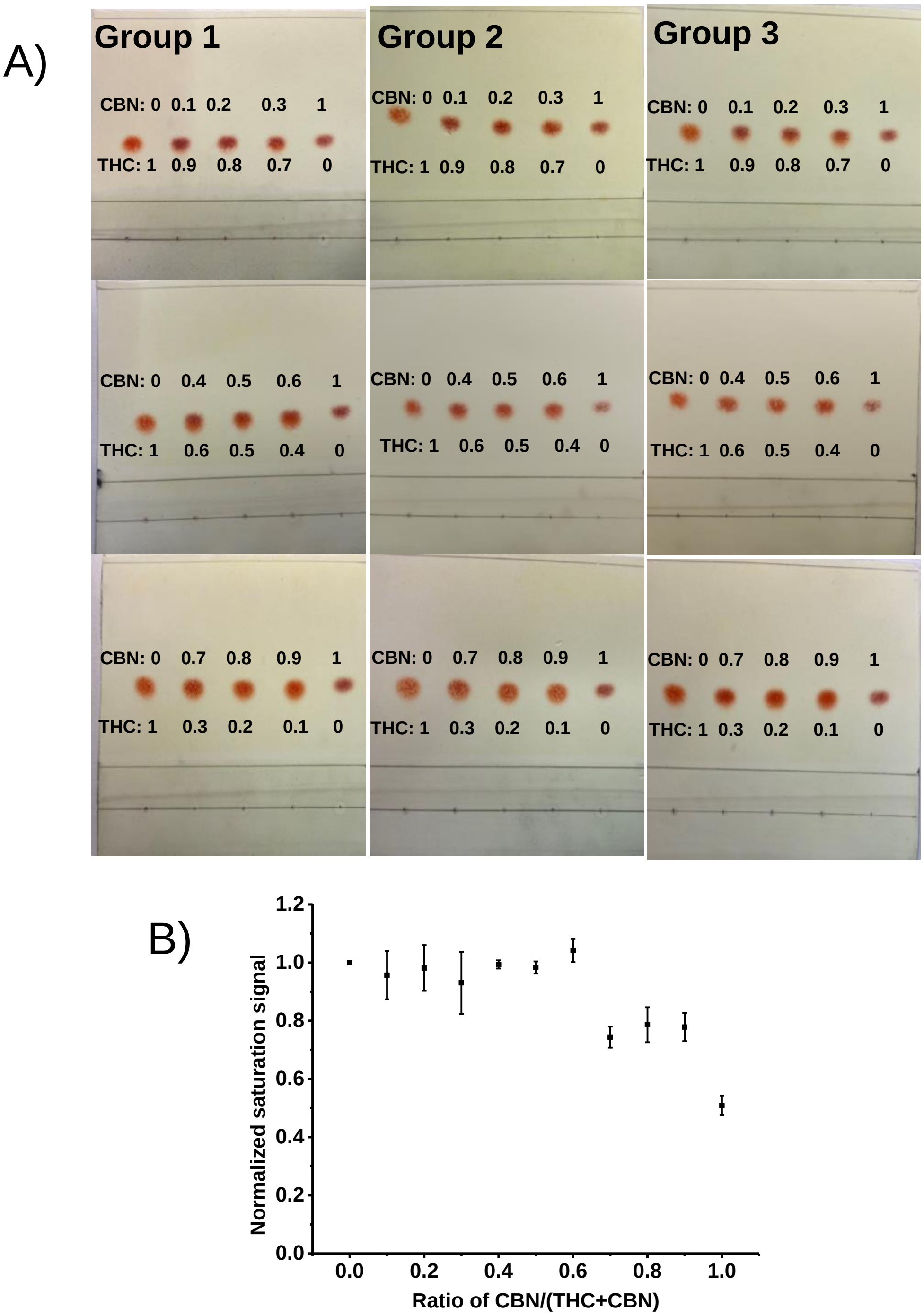

Figure S16. (A) Image of 1.00 μg (CBN+THC) with different CBN/THC ratio and (B) relationship between normalized saturation signal and composition of mixtures with different CBN/THC ratio, yet an equal total cannabinoid content. Error bars represent the standard deviation (n=3).

1.00 $\mu g \cdot \mu L^{-1}$ THC stock solution and 1.00 $\mu g \cdot \mu L^{-1}$ CBN stock solutions were mixed with different volumes to obtain eleven solutions. The volume of each solution was 10.0 μL, in which, the volume of THC stock solution was 0.0, 1.0, 2.0, 3.0, 4.0, 5.0, 6.0, 7.0, 8.0, 9.0 or 10.0 μL

and the volume of CBN stock solution was 10.0, 9.0, 8.0, 7.0, 6.0, 5.0, 4.0, 3.0,2.0, 1.0 or 0.0 μL. 1.0 μL of each of the above solutions was loaded on an Ag(I)-TLC plate, which was developed and analyzed. The smartphone saturation signal from each spot was divided by the saturation from pure THC saturation on the same plate for normalization. The obtained normalized saturation value was plotted against the CBN/(THC+CBN) ratio.

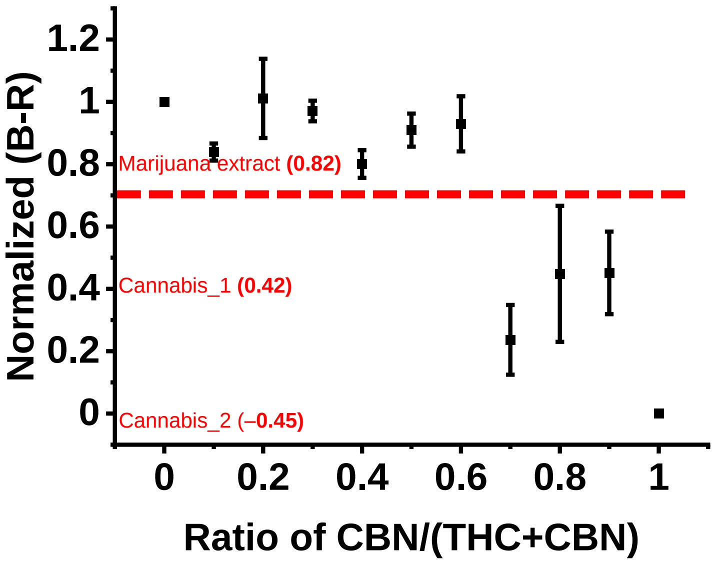

Figure S17. Relationship between normalized (B−R) signal and mixtures with different CBN/(THC+CBN) ratio.

ImageJ (NIH) software was then applied for image analysis of Figure S16A. First, the image was processed by automatically subtracting background with rolling ball radius 50.0 pixels. Next, a group of color spots with 1.0 μg THC spot on the left and 1.0 μg CBN spot on the right were selected by "straight line". Subsequently, RGB profile plot was obtained by performing "plugins→graphics→RGB profile plot". After that, independent R, G, B value of each spot were obtained by subtracting the independent R, G, B value of TLC background in between spots. Then, (B−R) of each spot was calculated and normalized between the (B−R) value for CBN (0) and THC (1). The obtained normalized (B−R) were plotted against THC/(THC+CBN) ratio to obtain Figure S17. Based on Figure S17, with a CBN/(THC+CBN) ratio larger than 0.6, the normalized (B−R) value is smaller than 0.7, which could be used as reference to determine whether to apply the THC calibration curve or CBN calibration curve to calculate the sum content of THC+CBN in samples.

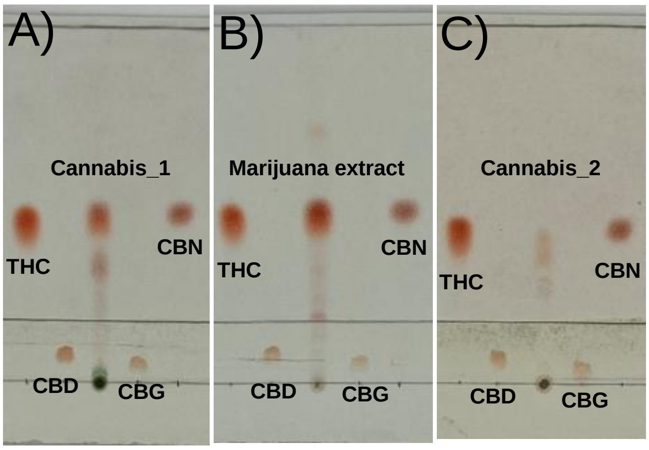

Figure S18. (A) Cannabis_1, (B) marijuana extract and (C) cannabis_2 accompanied with THC, CBN, CBD, CBG standards analyzed by Ag(I)-TLC.

RGB color analysis was performed on different cannabis samples according to the method described in Figure S17 and the obtained normalized (B−R) value were labelled in Figure S17.

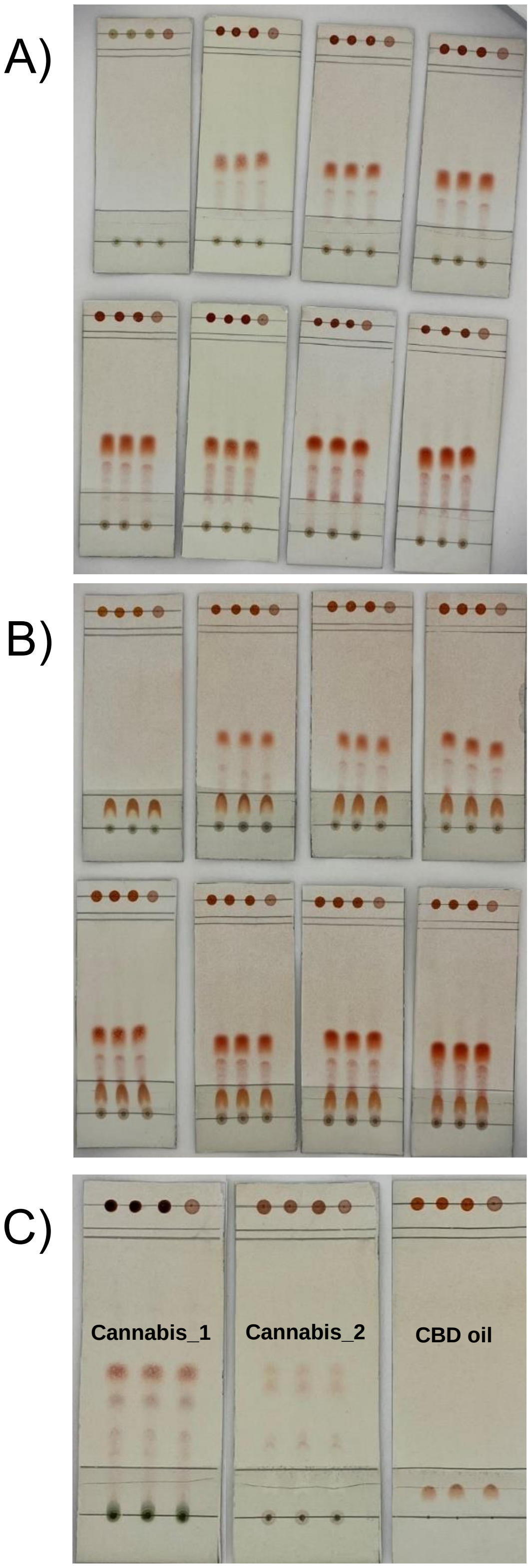

Figure S19. Images of (A) mixed cannabis extracts set I, (B) mixed cannabis extracts set II and (C) cannabis_1, cannabis_2 and CBD oil samples on Ag(I)-TLC.

ImageJ (NIH) software was applied for image analysis. Specifically, the image was converted to HSB stack type. Next, the targeted spot on the TLC plate was circled to measure

the integrated density value of the saturation. Saturation values of THC/CBN, THCA and olivetol spots were acquired accordingly. The THC/CBN and THCA values were then normalized against the olivetol reference value to correct for color development and photographing conditions. The normalized THC/CBN saturation value was used to calculate the sum content of THC+CBN in samples by the above constructed THC calibration curve or CBN calibration curve (Figure S14A or S14C). The normalized THCA saturation value was used to calculate the content of THCA in samples by the above constructed THCA calibration curve (Figure S14B). The calculated absolute amounts were divided by the sample loading volume (1 μL) to obtain concentrations. Subsequently, total THC potential was calculated as follows:

%THCA=[THCA]×(VOL/DW)×100;

%THC and %CBN were calculated similarly;

Total THC potential percentage=(%THCA×0.877)+%THC+%CBN;

[THCA]: measured concentration of THCA (μg/mL), VOL=external volume (300 μL), DW=dry sample weight (100.0 mg), 0.877=molecular weight ratio of cannabinoids to cannabinoid acids under study.

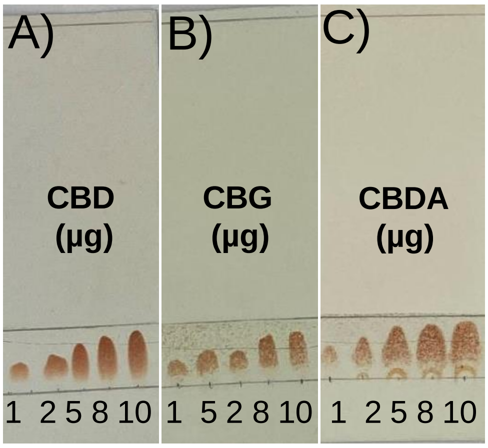

Figure S20. Various amounts of (A) CBD, (B) CBG and (C) CBDA standards analyzed by Ag (I)-TLC.

1.00 μg·μL^{-1} CBD stock solution, 1.00 μg·μL^{-1} CBG stock solution or 1.00 μg·μL^{-1} CBDA stock solution was loaded 1 time or repeatedly (2, 5, 8, or 10x) loaded after solvent evaporated to obtain an absolute loaded sample amount of 1.0, 2.0, 5.0, 8.0 or 10.0 μg. The Ag(I)-TLC plates were developed by solvent and then sprayed with color reagents.

References

(1) Baddiel, C. B.; Tait, M. J.; Janz, G. J. Nonaqueous Silver Nitrate Solutions. Spectral Studies in Acetonitrile. *J. Phys. Chem. C.* **1965**, 69, 3634-3638.

(2) McRae, G.; Melanson, J. E. Quantitative determination and validation of 17 cannabinoids in cannabis and hemp using liquid chromatography-tandem mass spectrometry. *Anal. Bioanal. Chem.* **2020**, *412*, 7381-7393.
